# A novel method for intelligent operation and maintenance of transformers using deep visual large model DETR + X and digital twin

**DOI:** 10.1038/s41598-024-83561-7

**Published:** 2025-01-02

**Authors:** Xuedong Zhang, Wenlei Sun, Ke Chen, Shijie Song

**Affiliations:** 1 School of Intelligent Manufacturing Modern Industry, Xinjiang University, Urumqi, 830046 Xinjiang China; 2 TBEA Co., Ltd., Changji, 831100 Xinjiang China

**Keywords:** Transformer peration and maintenance, Vision detection large model, Digital twin, Multi-modal, Multi-scale, Decision-making suggestions generation, Engineering, Electrical and electronic engineering, Information theory and computation, Techniques and instrumentation, Energy infrastructure, Power distribution, Power stations

## Abstract

To achieve real-time monitoring and intelligent maintenance of transformers, a framework based on deep vision and digital twin has been developed. An enhanced visual detection model, DETR + X, is proposed, implementing multidimensional sample data augmentation through Swin2SR and GAN networks. This model converts one-dimensional DGA data into three-dimensional feature images based on Gram angle fields, facilitating the transformation and fusion of heterogeneous modal information. The Pyramid Vision Transformer (PVT) is innovatively adopted as the backbone for image feature extraction, replacing the traditional ResNet structure. A Deformable Attention mechanism is employed to handle the complex spatial structure of multi-scale features. Testing results indicate that the improved DETR + X model performs well in transformer state recognition tasks, achieving a classification accuracy of 100% for DGA feature maps. In object detection tasks, it surpasses advanced models such as Faster R-CNN, RetinaNet, YOLOv8, and Deformable DETR in terms of overall mAP50 scores, particularly demonstrating significant enhancements in small object detection. Furthermore, the Llava-7b model, fine-tuned based on domain expertise, serves as an expert decision-making tool for transformer maintenance, providing accurate operational recommendations based on visual detection results. Finally, based on digital twin and inference models, a comprehensive platform has been developed to achieve real-time monitoring and intelligent maintenance of transformers.

## Introduction

Transformers are an indispensable component of power systems, widely used in power plants, substations, power grids, and various types of industrial and civilian facilities. They play critical roles in power transmission, energy distribution, and voltage regulation. In recent years, there has been a growing global focus on sustainable energy and environmental protection, with the advancement of new energy strategies such as carbon neutrality and peak carbon emissions, leading the international community to accelerate energy transition. As the “energy regulator” in the renewable energy sector and the “commander” of microgrids, the market demand for transformers has reached unprecedented levels. However, the state monitoring and maintenance tasks for transformers face significant challenges, necessitating more efficient and precise monitoring and maintenance techniques to ensure their healthy operation, thus guaranteeing the safety and stability of power systems.

In the field of transformer monitoring and maintenance, two of the most effective methods are dissolved gas analysis (DGA) based on transformer oil and infrared thermal images for hotspot detection. During abnormal operation of transformers, a series of chemical reactions occur due to reasons such as temperature rise and discharge, resulting in the generation of characteristic gases such as H_2_, CO, CO_2_, CH_4_, C_2_H_4_, C_2_H_6_, and C_2_H_2_. The generation rate and content ratio of these gases are correlated with the abnormal state of the transformer, providing a foundation for detecting transformer operating conditions based on DGA. Research on transformer operating condition monitoring based on DGA data can be categorized into traditional mechanistic analysis methods and artificial intelligence methods, according to their basic principles. Traditional mechanistic methods, including gas concentration alert method, gas growth rate alert method, characteristic gas method, three-ratio method, Duval triangle method, Dormenburg ratio method, Rogers ratio method, kernel density estimation method, conditional probability method based on multivariate Gaussian density estimation, and mathematical diagnostic methods based on fuzzy logic, are widely applied due to their simplicity, intuitive judgment, and ease of use^1,2^. However, the diagnostic accuracy and efficiency of transformer fault diagnosis based on such mathematical methods are limited. With the continuous development of artificial intelligence technology, various machine learning and deep learning algorithms have been extensively applied in the field of transformer fault recognition. Wani S A^3^ systematically elaborated existing methods and research progress based on DGA data analysis, indicating that the boundaries of traditional methods are inflexible and lack precision. Rajesh K N^4^ studied the impact of data balancing methods and machine learning techniques on the classification of transformer DGA faults, training various machine learning models based on DGA samples, with results demonstrating that carefully selecting data sampling methods and recognition algorithms is crucial for addressing DGA analysis issues. Rao U M^5^ identified that the high ambiguity, suitability, and model accuracy of DGA analysis methods pose key challenges in DGA fault classification, employing various machine learning algorithms for training, testing, and evaluation to identify the most suitable algorithm. Benmahamed Y^6^ enhanced the transformer fault diagnostic accuracy based on dissolved gas analysis (DGA) data with a proposed coupled system of support vector machine (SVM)-bat algorithm (BA) and Gaussian classifiers. Wang L^7^ conducted a comprehensive review and comparison of different enhancement methods for generating reliable data samples, aimed at balancing the diversity and distribution of DGA datasets. Zou D^8^ proposed a DGA analysis method based on Deep Belief Networks for diagnosing faults and conditions of power transformers with customized input features. Taha I B M^9^ introduced a Convolutional Neural Network (CNN) model based on DGA data analysis to accurately predict transformer fault types under varying noise levels. Zeng W^10^ proposed a CEEMDAN-DBN-ELM model, which employs Complete Ensemble Empirical Mode Decomposition with Adaptive Noise (CEEMDAN) to obtain multiple steady-state components. An improved Deep Belief Network (DBN-ELM) was then constructed to predict these multiple steady-state components. Additionally, infrared thermal imaging technology is widely applied in the industrial inspection domain, utilizing infrared cameras to detect the thermal distribution of transformer equipment in real time. This method allows for the rapid recognition of potential temperature anomalies without direct contact measurement, facilitating the detection of faults caused by overload, insulation damage, or other electrical issues. Research on transformer operational status monitoring based on infrared thermal imaging primarily involves capturing infrared images of various parts of the transformer using infrared cameras, and rapidly identifying abnormal hotspots’ locations and temperatures based on machine vision techniques. Mariprasath I^11^ constructed a real-time image acquisition system for monitoring transformer operational status using infrared thermal imaging technology. Xing Z^12^ proposed a two-phase multi-level threshold image segmentation method based on infrared images to complete the transformer fault diagnosis task. A.S. Nazmul Huda^13^ continuously acquired field images using an infrared camera and designed a preventive and predictive maintenance plan. Fanchiang K H^14^ utilized a thermal imaging monitoring system to collect normal and faulty images of transformers, training the Wasserstein Autoencoder Reconstruction (WAR) model and the Differential Image Classification (DIC) model to identify specific faults. The proposed models are characterized by their small storage size, fast inference time, and high accuracy. Fang J^15^ introduced a transformer fault diagnosis method based on infrared image processing and semi-supervised learning. The collected infrared image data underwent feature processing to extract temperature, texture, and shape features as model reference vectors, with a Generative Adversarial Network constructed to generate synthetic samples for a minority subset of labeled samples, followed by model training on the entire dataset. Li S^16^ researched a thermal imaging detection device based on infrared photoelectric sensors for fault detection of transformer bushing insulation. Chen L^17^ analyzed and simulated four typical overheating faults: turn-to-turn short circuits, airflow blockages, prolonged overload, and terminal overheating, establishing a fault infrared image dataset, and implemented accurate transformer fault detection based on an AlexNet model. Liu J^18^ proposed a contour-based instance segmentation network that achieved high-precision segmentation of high-voltage power equipment operation in complex and varying environments using infrared and visible light images.

In recent years, deep visual and digital twin technology have developed rapidly, emerging as hot topics in research and application across various fields. These advancements are expected to revolutionize the monitoring and maintenance practices for transformer equipment, introducing new means for efficient operation and maintenance. In the realm of visual inspection, deep visual technology effectively extracts information from images through a hierarchical structure, enabling the recognition and fusion of complex features. It has demonstrated remarkable performance in image classification, segmentation, and object detection tasks, achieving or even surpassing human recognition accuracy. Significant progress has been made in areas such as autonomous driving, medical imaging diagnosis, intelligent monitoring, behavior analysis and anomaly detection, as well as scene understanding and reconstruction in virtual reality and augmented reality. In addition to traditional Convolutional Neural Networks (CNN), a variety of deep visual detection models have emerged rapidly, with continuously evolving architectures and increasingly powerful computational capabilities. For instance, ResNet^19^ (Residual Network) addresses the gradient vanishing problem in deep networks by introducing skip connections, thereby enhancing the model’s training performance. The Inception^20^ network improves computational efficiency and accuracy by incorporating multi-scale convolutional kernels for parallel computation. Algorithms such as R-CNN, Fast R-CNN, Faster R-CNN, Mask R-CNN, and the YOLO series^21–25^ excel in object detection and image segmentation tasks, precisely localizing objects in images through Region Proposal Networks (RPN). Furthermore, Generative Adversarial Networks (GAN) have made significant advancements in image generation and data augmentation^26^. The application of Transformer^27^ models in the visual domain has also expanded, achieving improved global feature modeling capabilities through self-attention mechanisms. These algorithms collectively drive the in-depth development of deep visual technology across various application domains. Moreover, the research and application of digital twin technology have become increasingly widespread. With advancements and accumulation over recent years, the theoretical framework for digital twins has become complete, effectively integrating novel sensing technologies, data acquisition techniques, network analysis and transmission technologies, big data analytics, virtual reality, augmented reality, 3D visualization, and intelligent human–computer interaction. It merges concepts of cyber-physical systems, digital governance, and interdisciplinary knowledge, leading to more mature research and practice that has been widely applied in manufacturing, energy, healthcare, aerospace, and various other sectors. Applying digital twin technology to the monitoring and maintenance of transformer equipment can effectively meet the multidimensional control demands for remote real-time monitoring, virtual-real mapping, and virtual control of reality.

In summary, although a wealth of methods and techniques for monitoring and maintaining transformer equipment has been accumulated, the ongoing advancement of global renewable energy strategies poses significant challenges for the operation and maintenance of large-scale transformers in service. Traditional mechanistic analysis methods are inefficient and inaccurate, with a coarse granularity that no longer meets the current demands for high-quality, precise monitoring and analysis under complex operating conditions. Although many researchers have collected DGA data and infrared images from transformers and employed data analysis models or image detection models based on machine learning and convolutional neural networks(CNN) to identify transformer operating conditions, existing research methods have notable shortcomings that require further optimization and improvement. For instance, most studies rely solely on single-modal analyses of either DGA data or infrared images, lacking the capability to simultaneously incorporate information from both modalities, necessitating the establishment of multiple distinct models that cannot share information. Additionally, while the development of deep vision models has surged, with new architectures and models rapidly iterating, the computational performance of these models represents a significant improvement over traditional machine learning and CNN networks. However, there has been limited research and application of these new methods and theories in the monitoring and maintenance of transformer equipment. Furthermore, traditional monitoring and maintenance approaches for transformers exhibit poor real-time capabilities, failing to respond swiftly to anomalies in the operational environment, and lack robust virtual-physical integrated visualization for remote monitoring. To address these issues, this paper integrates deep vision technology and digital twin technology, proposing a real-time monitoring and intelligent operation and maintenance method for special transformers based on the DETR + X model. This approach constructs a virtual reality environment for remote monitoring and maintenance of transformers through digital twin technology, enabling a virtual-physical mapping and control system. Building on the state-of-the-art deep vision architecture DETR, an improved multi-modal, multi-scale vision detection model, DETR + X, has been designed. This model enhances the intelligent monitoring and maintenance of transformers through the integration and application of five modules: multi-dimensional data augmentation, multi-modal signal compatibility, multi-scale attention features, deformable attention mechanisms, and LLM-enhanced intelligent decision-making, aiming to elevate the level of intelligence and engineering applicability in transformer operation monitoring and maintenance.

## Materials and methods

### A digital twin-based monitoring and operation maintenance method for transformers using deep vision approaches

#### Basic structure and fault mechanism of transformers

**Fig. 1:**
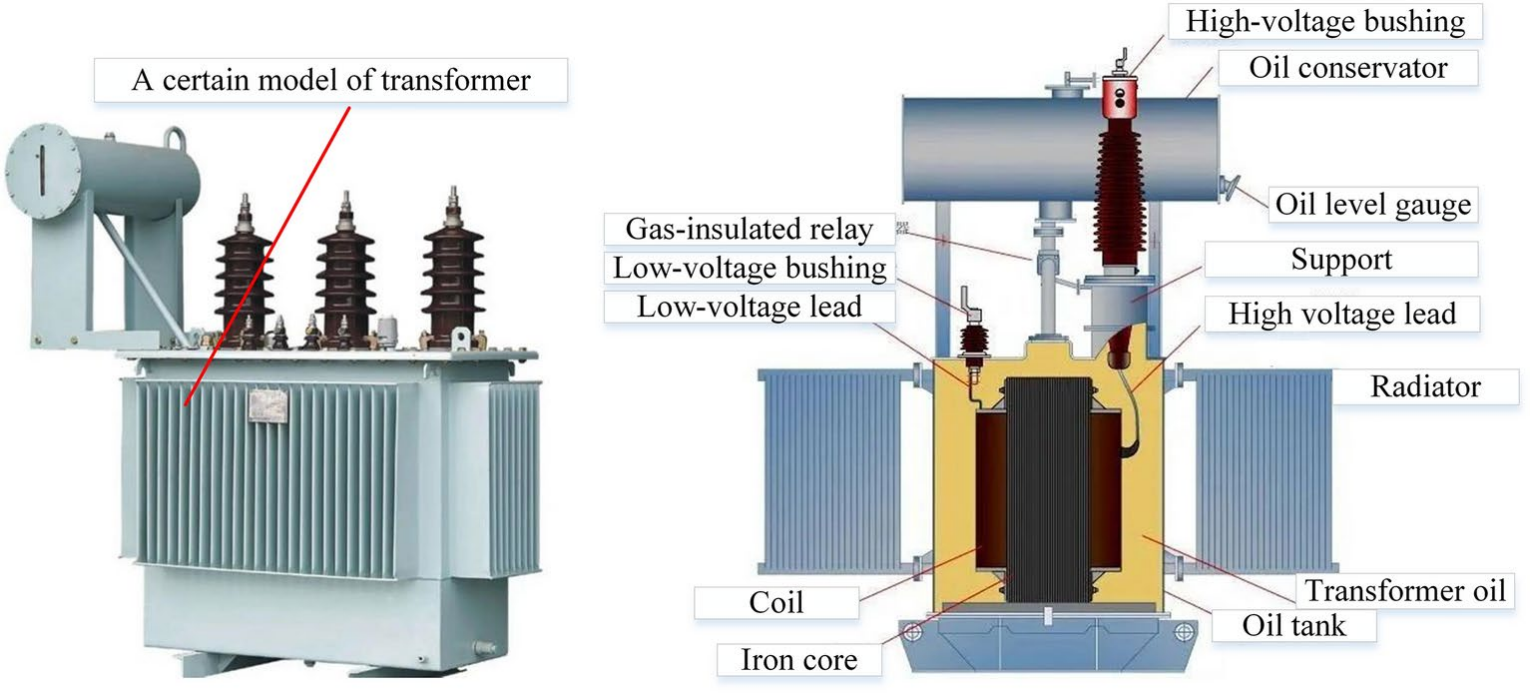
Basic Structure of a Transformer.

A transformer is a device that transmits electrical energy through the principle of electromagnetic induction, serving the functions of voltage transformation, current transformation, and impedance transformation. Despite the wide variety of transformer models, their substantial size differences, and diverse applications, their basic structure and operating principles are fundamentally the same. Taking a typical oil-immersed transformer as an example, it comprises several core components, including the iron core, windings, oil tank, cooling system, insulating bushing, and protective devices. The iron core forms the main magnetic circuit of the transformer and consists of two parts: the core pillars and the yoke. To enhance magnetic conductivity and reduce iron losses, the iron core is typically made of stacked hot-rolled or cold-rolled silicon steel sheets, with a thickness of 0.35–0.5 mm and coated with insulating paint. The windings constitute the electrical circuit of the transformer, generally made of insulated copper or aluminum wire (either flat or round). The primary winding is connected to the power supply, while the secondary winding is connected to the load. An alternating voltage is applied to the primary winding, generating alternating magnetic flux in both the primary and secondary windings, which induces electromotive force in each winding, thereby achieving voltage transformation. The basic structure of the transformer is illustrated in Fig. 1.

**Fig. 2:**
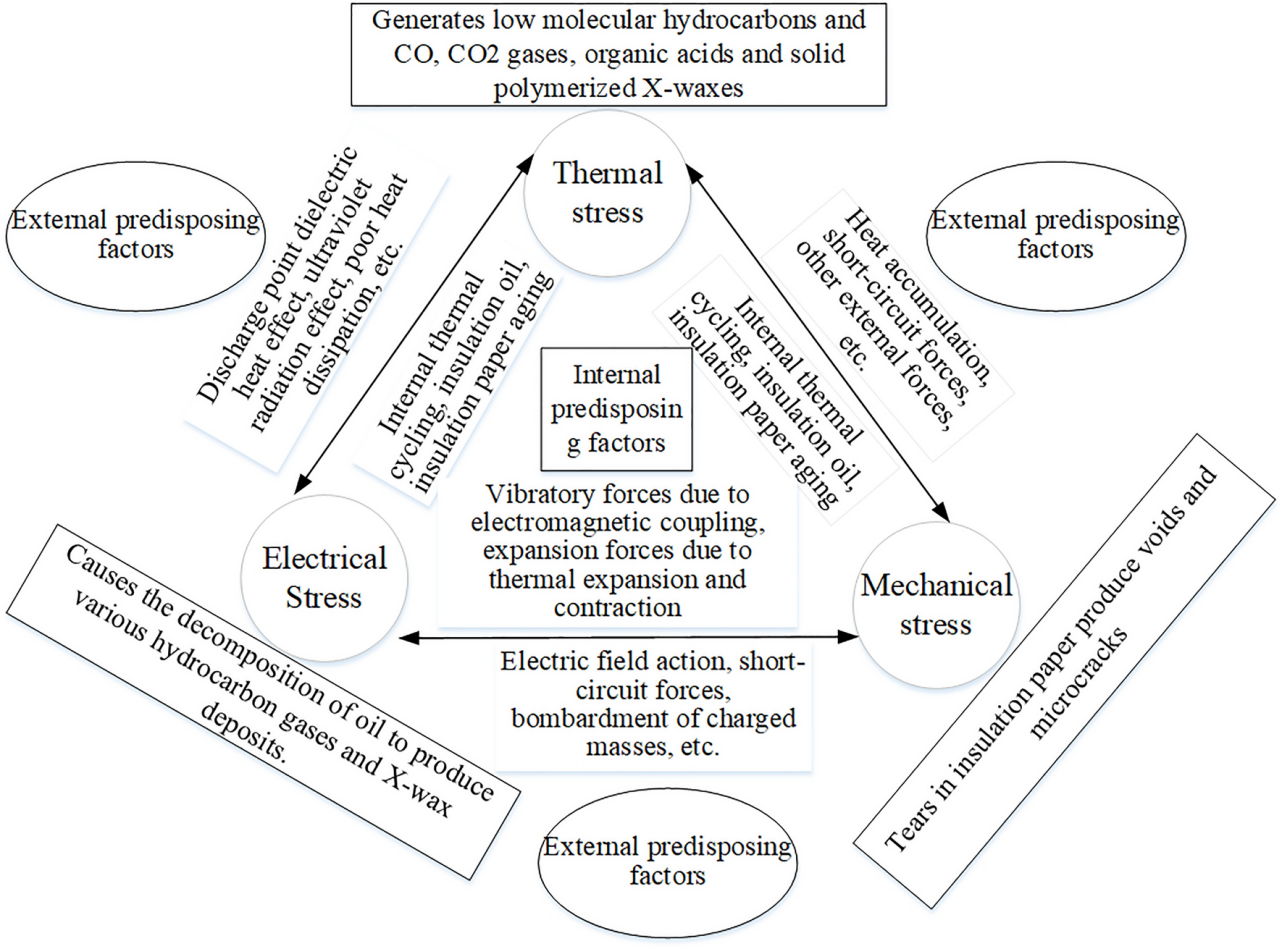
Fault Mechanisms of Transformers.

During the operation of transformer equipment, it is subjected to certain thermal, mechanical, and electrical stresses. These performance characteristics can be influenced by a range of external environmental disturbances as well as internal factors. For instance, external disturbances may include the effects of external electric fields, short circuits, bombardment by charged particles, and ultraviolet radiation, while internal factors encompass thermal cycling, aging of insulating paper and oil, vibrational forces due to electromagnetic coupling, and expansion forces resulting from thermal expansion and contraction, as illustrated in Fig. 2. When these stresses exceed normal thresholds, a series of anomalies and failures may occur in the operation of the transformer, such as abnormal heating in components like the radiator, high-voltage bushings, low-voltage bushings, cables, and electrical connection points, as well as partial discharge occurrences at air gaps, oil films, or conductor edges, alongside leakage of transformer oil. Transformers operating under abnormal conditions are often accompanied by a drastic rise in temperature, with heat continuously increasing and significantly exceeding the safe temperature range. Moreover, transformer insulating oil is a mixture composed of hydrocarbon molecules with varying molecular weights, under electrical and thermal faults, certain C-H and C–C bonds may break, resulting in the generation of a small quantity of reactive hydrogen atoms and unstable hydrocarbon radicals. Through complex chemical reactions, these hydrogen atoms or radicals can produce hydrogen gas and lower hydrocarbon gases. In the early stages of a fault, the gases formed dissolve in the oil, when the fault energy is significant, they accumulate as free gases, with varying gas contents indicating different abnormal operating states of the equipment. Furthermore, if the temperature at critical components and contact points rises abnormally, it may accelerate the electrochemical corrosion at the contact locations, leading to a vicious cycle of degradation, heating, and further degradation. Therefore, real-time monitoring and analysis of the abnormal heating phenomena in transformer equipment, the dissolved gases in the oil, and the leakage conditions of the oil are of great importance, as they can facilitate the timely detection of the abnormal operational status of transformers, thereby preventing the escalation of potentially general anomalies into severe faults.

#### A deep visual digital twin framework for monitoring and operation maintenance of transformers

**Fig. 3:**
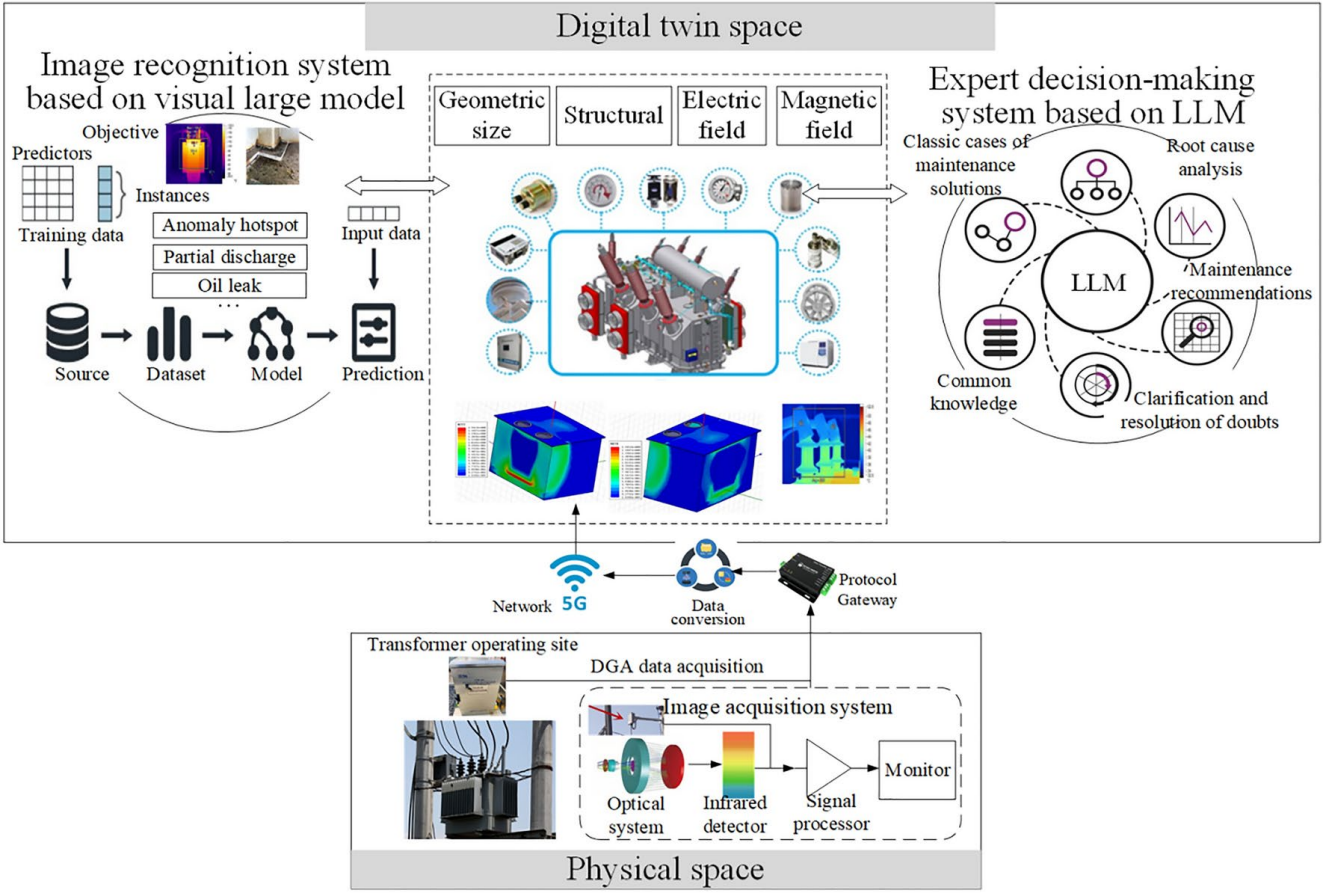
Architecture for Transformer Monitoring and Maintenance Based on Deep Vision and Digital Twin.

To enhance the precise and efficient monitoring and maintenance of abnormal states during transformer operation, this paper integrates deep visual inspection technology with digital twin, proposing a deep visual digital twin framework aimed at monitoring transformer operational states and facilitating intelligent maintenance, as illustrated in Fig. 3. This system constructs a virtual-reality fusion model for transformer operation, centered around deep visual detection tasks, based on the concept of digital twin. The model includes the geometric model, physical model, rule model, and data model of the transformer, enabling real-time monitoring, simulation, and intelligent analysis of the physical object. It efficiently perceives, maps, and analyzes issues such as abnormal heating, dissolved gas in oil, and oil leakage in transformers. This allows engineers to rapidly identify abnormalities in a non-contact manner without interrupting device operation. Specifically, infrared thermal imaging devices are employed for real-time temperature monitoring of critical transformer components (such as the body, cooling system, high-voltage bushing, low-voltage bushing, cables, and electrical connection points) to detect abnormal hotspots. Based on characteristic images corresponding to the values of dissolved gases in the oil, the system detects and analyzes six states: low energy discharge, high energy discharge, partial discharge, low-temperature overheating, medium-temperature overheating, and high-temperature overheating. Visible light images of the operational environment are utilized to recognize oil leakage of transformers. By utilizing digital twin technology to create a digital mapping of the physical entity of the transformer in the information space, the collected images are processed through deep vision algorithms to generate a virtual model that closely aligns with the physical object or operational state. This allows for real-time synchronous updates between the virtual model and the physical system, enabling the virtual environment to dynamically reflect changes in the physical world. Through key steps such as image acquisition, processing, and analysis, precise localization and classification of detection targets are conducted, thereby continuously updating the status of the digital twin model and achieving accurate monitoring and diagnosis of transformer operational states. Furthermore, based on the results, assistance and operational advice are sought from an LLM-based expert decision-making system, facilitating remote, efficient monitoring and intelligent maintenance of transformers.

### Proposed improved visual detection large model DETR + X

#### Basic idea of the DETR framework

**Fig. 4:**
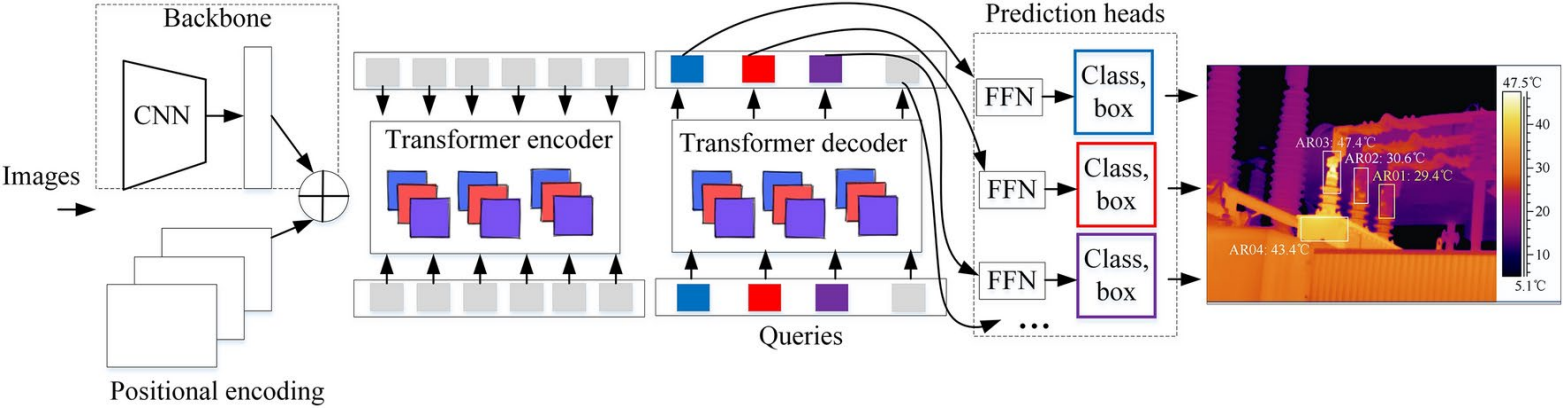
Basic Principles of the DETR Framework.

DETR (Detection Transformer)^28^ is an end-to-end object detection network based on the Transformer architecture, characterized by its Encoder-Decoder structure. The primary distinction from traditional convolutional networks is its elimination of the need for pre-defined anchor boxes and the Non-Maximum Suppression (NMS) post-processing strategy. The DETR model does not rely on complex manual designs or convolutional processes. It first employs a CNN backbone to extract features from the image, and then inputs the obtained features into a Transformer structure based on self-attention mechanisms, comprised of an Encoder and Decoder. After training, the model establishes a correspondence between specific queries and the image, allowing for the accurate prediction of bounding boxes and their corresponding classification labels. Compared to conventional detection frameworks, the DETR model boasts a simpler and more flexible architecture, enabling easy scalability and adaptability to various object detection tasks. It is particularly adept at capturing complex relationships within images, resulting in higher object detection accuracy and outperforming competitive baselines significantly in performance. The fundamental principles of the DETR framework are illustrated in Fig. 4.

#### Structure and principles of the improved DETR + X model

**Fig. 5:**
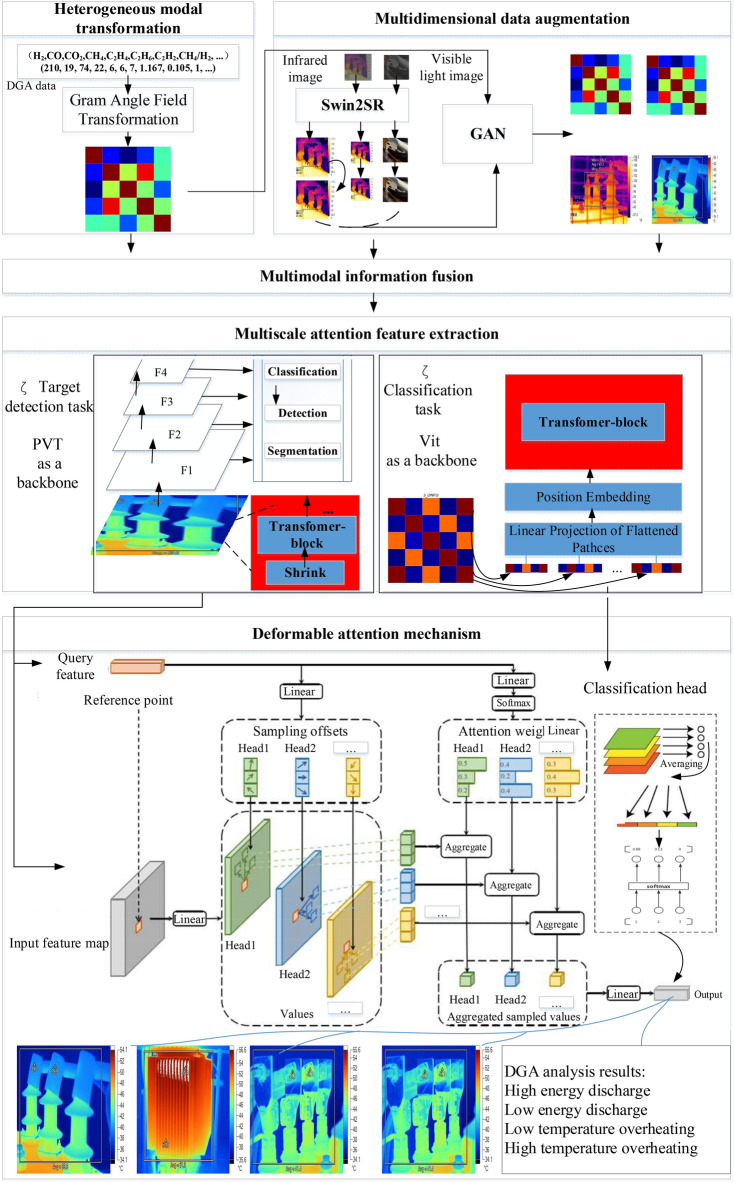
Overall Architecture of the Proposed DETR + X Model.

The operational environment of transformer equipment presents a diverse array of interference factors, including occlusion, dirt, corrosion, lighting, temperature, and humidity variations. This complexity in condition monitoring involves multiple modalities of data, such as DGA parameter values, infrared thermal images, and visible light images, which impose higher demands on the compatibility, accuracy, and engineering applicability of detection models. However, existing detection models still fall short of meeting these requirements, exhibiting several areas that need optimization and improvement. These issues are primarily reflected in the following aspects: (1) In real-world environments, the quality of transformer images captured by cameras is often unstable, with inconsistent sample scales and a limited number of samples under abnormal operating conditions. This leads to inadequate model training and learning, resulting in over fitting and poor generalization capabilities, thereby constraining the performance of object detection. (2) The use of convolutional networks such as ResNet as the backbone for feature extraction relies primarily on convolution operations to capture local features of images. While multi-layer convolutions can accumulate more global information by increasing the receptive field, this approach still depends on local computations, resulting in weak global modeling capabilities. This limitation is particularly evident in scenarios requiring long-range dependencies, such as detecting small or sparse objects in complex scenes. Furthermore, although ResNet employs multi-layer feature maps to represent information at different scales, its scale granularity is insufficient, especially when addressing targets with significant size variations in large scenes, leading to limited multi-scale processing capabilities and a tendency to overlook small objects or fine-grained features. (3) Most existing transformer condition recognition models are based on single modalities, which do not adequately accommodate the input and computation of multi-modal data. This necessitates the construction of multiple single-modal detection models running in parallel, which not only increases the number and complexity of models but also hinders the integration and sharing of multi-modal information. (4) In current research, while anomaly detection and fault diagnosis models have achieved a degree of success in predicting transformer states, they do not provide operational decision-making suggestions based on the predictive results. Furthermore, there is a lack of in-depth analysis of the root causes of anomalies and faults based on specialized knowledge, making it difficult to guide equipment managers in preventive detection and maintenance. To address these issues, this paper builds upon the state-of-the-art DETR model architecture and focuses on research and exploration in areas such as multi-dimensional data augmentation, multi-modal information fusion, multi-scale feature extraction, and LLM-enhanced operational decision-making. We propose an improved multi-modal, multi-scale vision detection model, DETR + X, with an overall architecture illustrated in Fig. 5.

##### Multi-dimensional data augmentation

**Fig. 6:**
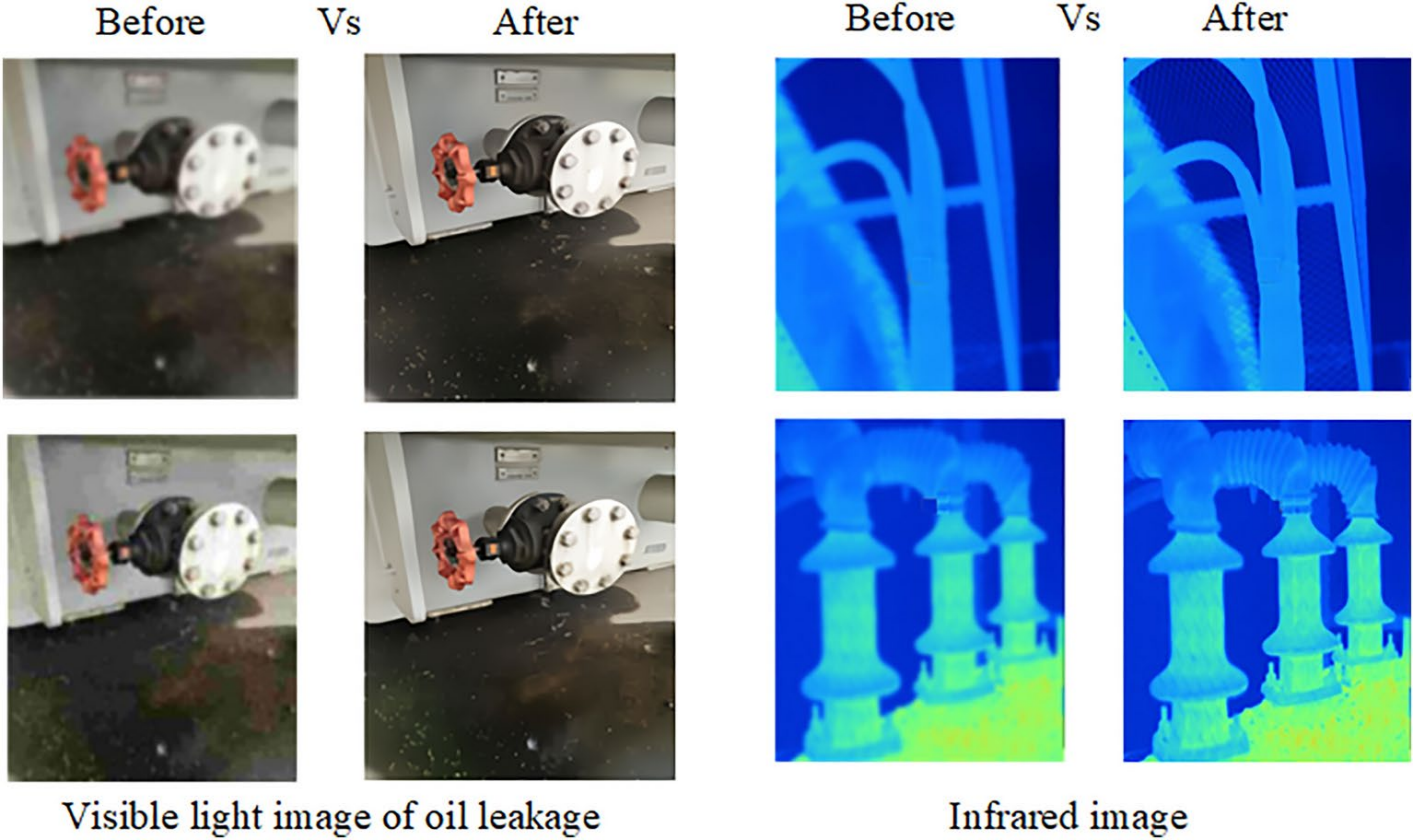
High-Quality Sample Reconstruction Based on Swin2SR.

In terms of data augmentation, the Swin2SR model is employed to optimize the collected raw images in order to enhance their quality. By leveraging its global context-awareness and self-attention mechanism, the model accurately captures both local and global features of the images. Through the learning of mappings between high and low-resolution images, it reconstructs image details and enhances the representation of complex textures and edge regions, thereby establishing a data foundation that improves the model’s generalization capabilities. The augmented images are shown in Fig. 6, where the overall noise in the samples is significantly reduced, while brightness, edges, and contrast are more pronounced, resulting in better resolution. Additionally, the subtle details of the samples are more prominent, leading to a marked improvement in overall image quality.

At the same time, DETR is a large model framework designed for visual detection, and training it on a large-scale dataset can fully leverage the advantages of the model architecture to achieve better performance. However, the number of image samples in abnormal situations is often limited. To expand the scale of the training dataset, GAN (Generative Adversarial Network) is employed to conduct adversarial training between a generator and a discriminator, allowing the model to learn the data characteristics and distribution of real samples. This process generates realistic augmented samples, thus assisting the model in learning a broader range of data features at the dataset level, which enhances the robustness of the model in practical applications. Below is a formal description of the multi-dimensional data augmentation process.

Assume that the original collected image dataset contains n samples, where *X*_i_ represents the three-dimensional RGB pixel matrix and its annotation information for each sample. The original dataset can be expressed as:

1 $$D = \{ x_{1} ,x_{2} ,...,x_{n} \}$$

Define the function *f*_Swin2SR_ as the data augmentation operation based on the Swin2SR model. This function processes each image *X*_*i*_ by applying denoising, high-resolution reconstruction, and other operations to generate a high-quality augmented dataset, described as:

2 $$\mathop {x_{i} }\limits^{\sim } = f_{Swin2SR} (x_{i} )$$

3 $$\mathop D\limits^{\sim } = \{ \mathop {x_{1} }\limits^{\sim } ,\mathop {x_{2} }\limits^{\sim } ,...,\mathop {x_{n} }\limits^{\sim } \}$$

Define the function *f*_GAN_ as a sample size expansion generation operation based on the GAN network. Through adversarial learning and training of the generator and discriminator, it accurately simulates the distribution of real data, generating a large number of samples that closely resemble real data:

4 $$x_{j}^{\prime } = f_{GAN} \left( {z_{j} } \right)$$

Here,* X*_*j*_ represents new samples generated from random vectors in the latent space (following a Gaussian distribution), and the expanded dataset is represented as:

5 $$D{\prime} = \{ x_{1} ^{\prime},x_{2} ^{\prime},...,x_{m} ^{\prime}\}$$

The final high-quality expanded dataset used for model training is denoted as:

6 $$\mathop D\limits^{ \wedge } = \mathop D\limits^{\sim } \cup D^{\prime} = \{ f_{Swin2SR} (x_{1} ),f_{Swin2SR} (x_{1} ),...,f_{Swin2SR} (x_{n} )\} \cup \{ f_{GAN} (x_{1} ),f_{GAN} (x_{1} ),...,f_{GAN} (x_{n} )\}$$

##### Multimodal information fusion

In existing research, DGA data analysis and infrared image detection require different models. DGA data analysis is a classification and prediction problem based on the gas content values in the oil, while infrared image detection is a target detection problem based on images. To construct a unified monitoring model for transformer operating conditions, this paper proposes a method for modal transformation of DGA data based on the Gram angle field theory. The Gram angle field is a mapping that transforms one-dimensional waveforms into two-dimensional images in Cartesian coordinates, reflecting the correlation among various gas parameters in the DGA data. Transforming DGA data into images facilitates the subsequent image classification model’s ability to extract the chromatographic information of the oil. To begin with, the step size and amplitude in the DGA data are transformed into radius and angle through polar coordinate projection. After that, the correlation between each point in polar coordinates is measured using trigonometric functions, forming a Gram matrix through trigonometric transformation. The specific process of heterogeneous modal transformation based on the Gram angle field is as follows:

For a given DGA data entry: $X = \{ x_{1} ,x_{2} ,...,x_{i} ,...,x_{n} \}$, $x_{i}$ representing a specific dissolved gas component in the transformer oil, normalization is first applied, resulting in a new data value of:

7 $$x_{i}{\prime} = \frac{{[(x_{i} - \max X) + (x_{i} - \min X)]}}{\max X - \min X}$$

Subsequently, the normalized $x_{1}{\prime}$ is mapped to the polar coordinate system, with the magnitude encoded as the cosine angle $\theta$, $\theta \in [0,\Pi ]$, preserving the numerical relationship. Additionally, the order is mapped to the radius *r*, maintaining the spatial relationship. The mapping relationship is expressed as follows:

8 $$\left\{ \begin{gathered} \theta_{i} = \arccos x_{i} ^{\prime}, - 1 \le x_{i} ^{\prime} \le 1,x_{i} ^{\prime} \in X^{\prime} \hfill \\ r_{i} = \frac{i}{n},1 \le i \le n \hfill \\ \end{gathered} \right.$$

where, $\theta_{i}$ represents the polar angle at point* i*, while $r_{i}$ denotes the polar radius at point* i*.

Within the interval $\theta \in [0,\Pi ]$, the function $\cos \theta$ is monotonically decreasing, ensuring a unique corresponding result in polar coordinates for a given sequence. Furthermore, the polar radius r guarantees an absolute temporal relationship, constituting a bijective transformation. Based on the aforementioned polar coordinates, the Gram angle and Gram angle difference fields are calculated using Eqs. (9) and (10):

9 $$GASF = \left\{ \begin{gathered} \cos (\theta_{1} + \theta_{1} )...\cos (\theta_{1} + \theta_{j} ) \hfill \\ \cos (\theta_{2} + \theta_{1} )...\cos (\theta_{2} + \theta_{j} ) \hfill \\ \vdots \hfill \\ \cos (\theta_{i} + \theta_{1} )...\cos (\theta_{i} + \theta_{j} ) \hfill \\ \end{gathered} \right\}$$

10 $$GADF = \left\{ \begin{gathered} \sin (\theta_{1} + \theta_{1} )...\sin (\theta_{1} + \theta_{j} ) \hfill \\ \sin (\theta_{2} + \theta_{1} )...\sin (\theta_{2} + \theta_{j} ) \hfill \\ \vdots \hfill \\ \sin (\theta_{i} + \theta_{1} )...\sin (\theta_{i} + \theta_{j} ) \hfill \\ \end{gathered} \right\}$$

**Fig. 7:**
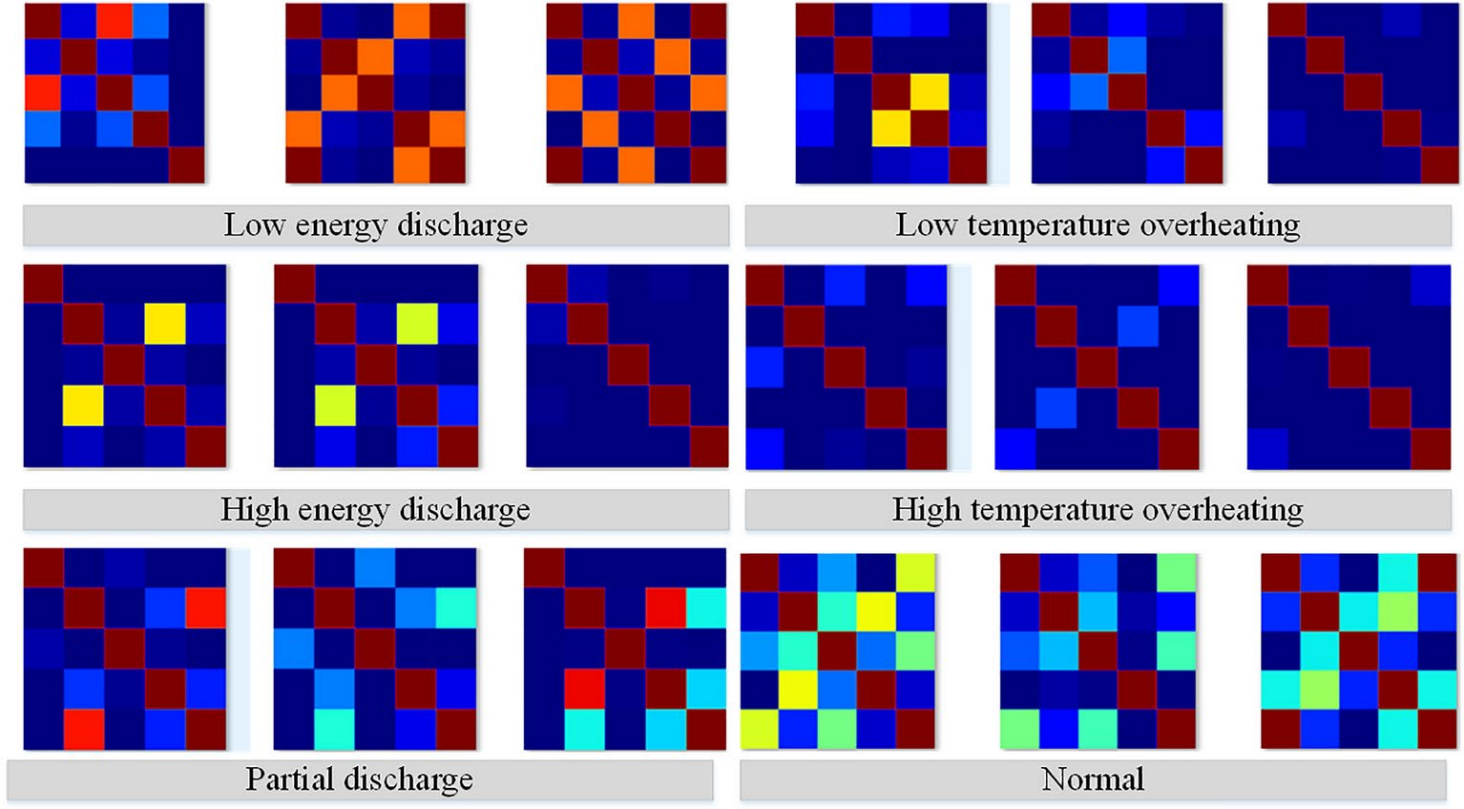
Generation of RGB Feature Maps from DGA Data Based on Gram Angle Fields.

Formula (9) specifies the calculation method for the Gram angle field, while formula (10) outlines the calculation method for the Gram angle difference field, where $\theta_{j}$ represents the polar angle at point *j*. Finally, the results are converted into a standard RGB image, serving as the feature map for the DGA data. As shown in Fig. 7, the Gram angle field feature maps generated from the DGA data illustrate different fault categories.

##### Multi-scale attention features

The existing DETR model employs CNN networks (such as ResNet) as its feature extraction backbone, primarily using different convolution strides to obtain multi-scale feature maps. In contrast, the Pyramid Vision Transformer (PVT) adopts a progressive compression strategy, controlling feature map scales through layer embedding. Relevant research indicates that the PVT model achieves better performance with the same parameters and computational overhead^30^. Therefore, in this study, the DETR + X model replaces the original ResNet backbone with the Pyramid Vision Transformer (PVT) as the new backbone. This integration introduces a pyramid structure into the Transformer, further enhancing the model’s ability to express multi-scale features. Relevant parameters of the Pyramid Vision Transformer (PVT) model are shown in Table 1.

**Table 1:** Parameter Settings for the Pyramid Vision Transformer (PVT).

Feature stage	Output feature map scale (original input H*W)	Number of channels	Patch size	SRA reduction ratio	Number of heads	Feed forward layer expansion rate
S1	H/4 *W/4	64	4*4	8	1	8
S2	H/8 *W/8	128	2*2	4	2	8
S3	H/16 *W/16	320	2*2	2	5	4
S4	H/32 *W/32	512	2*2	1	8	4

##### Deformable attention mechanism

The traditional attention mechanism requires calculation of the relevance between each query and all global positions, which significantly increases computational complexity and spatial dimensions while primarily focusing on global information. However, this approach has limited capacity for perceiving local details. To address such issues, researchers have proposed a novel attention mechanism known as Deformable Attention. Deformable Attention operation allows for the adaptive selection of a small number of reference points for attention calculation through operations such as reference point generation, sampling position and offset computation, attention computation, and result aggregation. This mechanism can more effectively focus on key regions within an image, offering higher computational efficiency and flexibility, making it particularly suitable for tasks requiring dynamic attention to local areas. Furthermore, the attention mechanism based on the Transformer architecture is inherently adept at capturing long-range dependencies and provides a more comprehensive representation for the fusion of global features. With this mechanism, the model possesses exceptional learning capabilities for both local and global features. Additionally, it can dynamically adjust the shape and size of the attention model to better accommodate the characteristics of different tasks and input data, significantly reducing computational load. Compared to standard self-attention mechanisms and Convolutional Neural Networks (CNNs), Deformable Attention exhibits faster convergence rates and learning capabilities. Therefore, this study employs the Deformable Attention mechanism in the DETR + X model. The multi-scale attention features extracted by the Pyramid Vision Transformer (PVT) are input into the Deformable Attention module for training and inference, thereby flexibly handling more complex spatial structural information to achieve robust learning and reasoning capabilities.

##### LLM-enhanced intelligent decision-making for operation and maintenance

Existing research primarily focuses on identifying anomalies and issues during transformer operation. However, once a fault alert is detected, on-site maintenance personnel still need to consult expert engineers. These experts assess the severity of the issue based on their experience and provide repair and maintenance solutions. This approach leads to excessive operational response times and continues to rely on human involvement, which limits the efficiency of operations and maintenance and results in a low level of automation. Therefore, this study aims to achieve end-to-end integration of the transformer anomaly detection task based on large visual models with intelligent decision-making maintenance tasks. Historical decision data related to transformer operation and maintenance, expert knowledge, and repair cases are utilized as corpora to fine-tune the LLM (this study employs the LLAVA_7b model), resulting in an intelligent operation and maintenance expert decision system capable of facilitating human–machine and machine-machine dialogues. The inference results from the visual large model are organized into textual descriptions and input into the LLM. The LLM then performs reasoning analysis based on the described anomalous state of the transformer, explaining the root causes of the anomalies and generating actionable maintenance decision recommendations to guide the maintenance personnel in their tasks.

## Results

### Dataset construction

The data utilized in this study primarily consists of three types of data: DGA data, infrared thermal imaging, and leaked oil images. The original DGA data includes six failure categories: partial discharge, low energy discharge, high energy discharge, low-temperature overheating, medium-temperature overheating, and high-temperature overheating. To adapt to the model input, Gram angle fields are used to transform one-dimensional DGA samples into corresponding three-dimensional RGB feature maps. The infrared images of the transformer encompass annotations for components such as the body, heat dissipation structures, high-voltage bushings, low-voltage bushings, cables, and electrical connection points, as well as hot spot coordinates. Additionally, the positions of oil leaks in visible light images are annotated. Based on the Swin2SR and GAN data augmentation methods proposed in this study, the original images are processed for denoising, high-resolution enhancement, and detail reconstruction, resulting in a scaled-up training dataset for model training. The composition of the transformer dataset is illustrated in Table 2.

**Table 2:** Constructed Transformer Dataset.

Sample type	Types of anomalies or faults	Location	Data sample
DGA	Low energy discharge	/	The original data is a one-dimensional vector composed of DGA gas content, which has been converted into RGB feature map:
	High energy discharge	/	
	Partial discharge	/	
	Low temperature overheating	/	
	Medium temperature overheating	/	
	High temperature overheating	/	
Infrared image	Anomalous hotspot	Radiator	
		High voltage bushing	
		Low voltage bushing	
		Cable	
		Electrical connection point	
		Body	
Oil leakage image	Oil leakage	Oil leakage Shadow …	

Additionally, to comprehensively compare and validate the performance of the model proposed in this paper, several other publicly available datasets were prepared for evaluation under the same benchmarking criteria. These primarily include the NEU Surface Defect Database (NEU-CLS) provided by Northeastern University, the FLIR Thermal Dataset released by FLIR Systems, and the Printed Circuit Board Defect Dataset (PCB) jointly published by Shenzhen University and Xiamen University. A brief overview of these datasets is provided below:

FLIR Thermal Dataset: Released by FLIR, this infrared image dataset includes various types of infrared images and visible light images. The dataset was obtained using an RGB camera and a thermal imaging camera, encompassing a total of 14,452 infrared images. It is widely used in applications such as image fusion and object detection.

PCB Dataset: This dataset is designed for the detection and classification of defects on printed circuit boards (PCB). It contains 1,386 images along with six types of defects (missing holes, mouse bites, open circuits, short circuits, spurious, and false copper), and it is used for detection and classification tasks.

NEU-CLS Dataset: This dataset is intended for surface defect detection and contains images of six typical surface defects. It is primarily utilized for classification and localization tasks, comprising six types of typical surface defects found in hot-rolled steel strips, including rolling scale, spots, cracks, pitting surfaces, inclusions, and scratches. Each defect category contains 300 samples, totaling 1,800 grayscale images.

### Model training and evaluation

#### Main results

To thoroughly validate the effectiveness of the proposed model in this paper, training and testing were conducted on the transformer dataset (including DGA feature maps, infrared hotspot images, and oil leakage images) as well as several other publicly available datasets. This involved the use of the YOLOV8 model based on convolutional networks, and visual detection models based on Transformer architecture (such as the standard DETR model, Deformable DETR model, and the improved model DETR + X). The results are presented in Tables 3, 4 and 5.

**Table 3:** Comparison of Different Algorithms’ Performance on Object Detection Tasks in Public Datasets.

Dataset	Algorithm	Epoch	mAP50	AP_small	Remarks
FLIR Thermal	Faster-RCNN	100	0.408	0.173	20% Sparse sampling
	YOLOV8	100	0.429	/	
	Retinanet	100	0.232	0.085	
	Deformable_DETR	50	0.450	0.185	
	Deformable_DETR	100	0.510	0.230	
	DETR + X	50	0.460	0.200	
	DETR + X	100	0.532	0.262	
PCB	Faster-RCNN	100	0.758	0.601	/
	YOLOV8	100	0.970	/	/
	Retinanet	100	0.927	0.601	/
	Deformable_DETR	50	0.962	0.751	Backbone: ResNet50
	Deformable_DETR	100	0.973	0.759	
	DETR + X	50	0.977	0.770	Backbone: PVT_V2_B2
	DETR + X	100	0.985	0.781	
NEU-CLS	Faster R-CNN	100	0.665	0.352	/
	YOLOV8	100	0.759	/	/
	Retinanet	100	0.689	0.321	/
	Deformable_DETR	50	0.650	0.255	Backbone: ResNet50
	Deformable_DETR	100	0.670	0.351	
	DETR + X	50	0.665	0.404	Backbone: PVT_V2_B2
	DETR + X	100	0.722	0.463	
COCO	DETR	500	0.420	0.205	This data is cited from reference^29^
	Deformable_DETR	50	0.445	0.271	
	Faster R-CNN + FPN	109	0.420	0.266

**Table 4:** Comparison of Different Algorithms’ Performance on Object Detection in Transformer Datasets.

Dataset	Algorithm	Epoch	mAP50	AP_small	Remarks
Infrared images of the transformers	YOLOV8	100	0.371	/	/
	Faster R-CNN	100	0.510	0.243	/
	Retinanet	100	0.495	0.262	/
	Deformable_DETR	50	0.613	0.260	Backbone: ResNet50
	Deformable_DETR	100	0.653	0.270	
	DETR + X	50	0.629	0.303	Backbone: PVT_V2_B2
	DETR + X	100	0.713	0.344	
Oil leakage image of the transformers	YOLOV8	100	0.612	/	/
	Faster R-CNN	100	0.800	0.373	/
	Retinanet	100	0.906	0.413	/
	Deformable_DETR	50	0.897	0.237	Backbone: ResNet50
	Deformable_DETR	100	0.925	0.350	
	DETR + X	50	0.955	0.324	Backbone: PVT_V2_B2
	DETR + X	100	0.975	0.450

**Table 5:** Comparison of Different Algorithms’ Performance on Classification Tasks in DGA Data.

Method type	Algorithm	Data type	Accuracy (%)	Remarks
Mechanistic model	Non-parametric kernel density	Raw Data of DAG	64	/
	Gaussian density	Raw Data of DAG	54.4	/
	Fuzzy set	Raw Data of DAG	53.8	/
Machine learning	Random Forest	Raw Data of DAG	88	/
	AdaBoost	Raw Data of DAG	56	/
	KNN	Raw Data of DAG	65	/
	BP neural network	Raw Data of DAG	40	/
	Logistic regression	Raw Data of DAG	67	/
Deep network	CNN	DGA Feature Image	81	Epoch = 5
	CNN	DGA Feature Image	95	Epoch = 10
	EfficientNet-B0	DGA Feature Image	98	Epoch = 5
	ResNet-18	DGA Feature Image	98	Epoch = 5
	DETR + X	DGA Feature Image	100	Epoch = 5 (The classifier is Vit)

As shown in Table 3, previous research has demonstrated that the Deformable DETR model achieves higher mAP50 scores and better small object detection capabilities compared to the standard DETR and Faster R-CNN + FPN on the COCO dataset^29^. The performance difference between Deformable DETR and the standard DETR can be attributed to the different attention mechanisms used in the two models. DETR employs a standard attention mechanism, while Deformable DETR utilizes a deformable attention mechanism. This result suggests that the deformable attention mechanism may be effective in enhancing the model’s object detection capabilities.

In this experiment, the most representative algorithms, which are widely recognized as some of the best image object detection algorithms in existing research—such as Faster R-CNN, YOLOv8, and RetinaNet—were selected for comparative analysis with the proposed improved DETR + X model. The results indicate that the proposed improved DETR + X model achieves leading mAP50 scores and demonstrates significantly enhanced performance in small object detection, clearly outperforming other models. Specifically, on the FLIR Thermal and PCB datasets, the Deformable DETR and DETR + X models require fewer epochs than other models to approach or surpass their best mAP50 scores. Under the same number of epochs, the DETR + X model proposed in this study consistently achieves higher mAP50 scores than other models, reaching optimal performance. It is worth noting that, in the FLIR Thermal dataset, the improved DETR + X model achieved the best performance using only 20% of the sparse samples for training. This suggests that the DETR + X model has superior generalization ability, effectively handling unseen samples that slightly differ from the training data, and making accurate predictions. On the NEU-CLS dataset, the proposed improved DETR + X model, after multiple training sessions, achieves mAP50 scores close to the state-of-the-art YOLOv8 model (although it has not yet surpassed YOLOv8). The reason for this result may primarily lie in the lower resolution of the NEU-CLS dataset, along with the simple and regular shapes of the samples, uniform and consistent backgrounds, and the lack of prominent global features in the images. As a result, the global joint modeling advantage of the DETR + X model in handling complex scenes and long-range dependencies has not been fully realized. Nevertheless, the DETR + X model still demonstrates strong object detection capabilities.

In summary, the DETR + X model proposed in this study outperforms other state-of-the-art methods in terms of overall performance. On the same datasets, the improved DETR + X model achieves higher mAP50 scores compared to its predecessor. Additionally, it excels in small object detection, almost reaching the best results among other models.

As shown in Table 4, in the object detection task on the transformer dataset constructed in this study, both the Deformable DETR and DETR + X models outperform the YOLOv8, Faster R-CNN, and RetinaNet models by a significant margin. Moreover, DETR + X achieves impressive performance with fewer epochs, showcasing faster convergence. The improved DETR + X model demonstrates a clear advantage over the pre-improvement model in both mAP50 scores and small object detection performance. This indicates that the DETR + X model proposed in this study has significant advantages in transformer infrared image detection and oil leak detection, making it both effective and advanced.

Additionally, to validate the performance of the improved DETR + X model in classification tasks on the DGA dataset, experiments were conducted using various traditional mechanistic models, machine learning models, deep convolutional networks, and the Vision Transformer (ViT) model used by DETR + X. The results, as shown in Table 5, indicate that the classification accuracy of machine learning and mechanistic models is significantly lower than that of models based on deep networks. Among the models that classify images using DGA feature maps as input, EfficientNet-B0 and ResNet-18 achieved an accuracy of 98%, while the fine-tuned pre-trained ViT model reached a classification accuracy of 100% in just a few epochs. This indicates that the approach of converting DGA data into feature images based on the Gram angular field algorithm, combined with the advanced visual classification backbone, effectively achieves the highest accuracy in DGA data analysis.

#### Ablation study

To further explore the effectiveness and significant contributions of the key components in the proposed method of this paper, extensive ablation experiments were conducted on the following aspects: data augmentation based on Swin2SR, multi-scale attention based on Pyramid Vision Transformer (PVT), deformable attention mechanism, DGA data modality transformation, and the intelligent decision-making module based on LLM. A systematic comparison and analysis of the importance and effectiveness of these components were performed.

##### Data augmentation based on Swin2SR

**Fig. 8:**
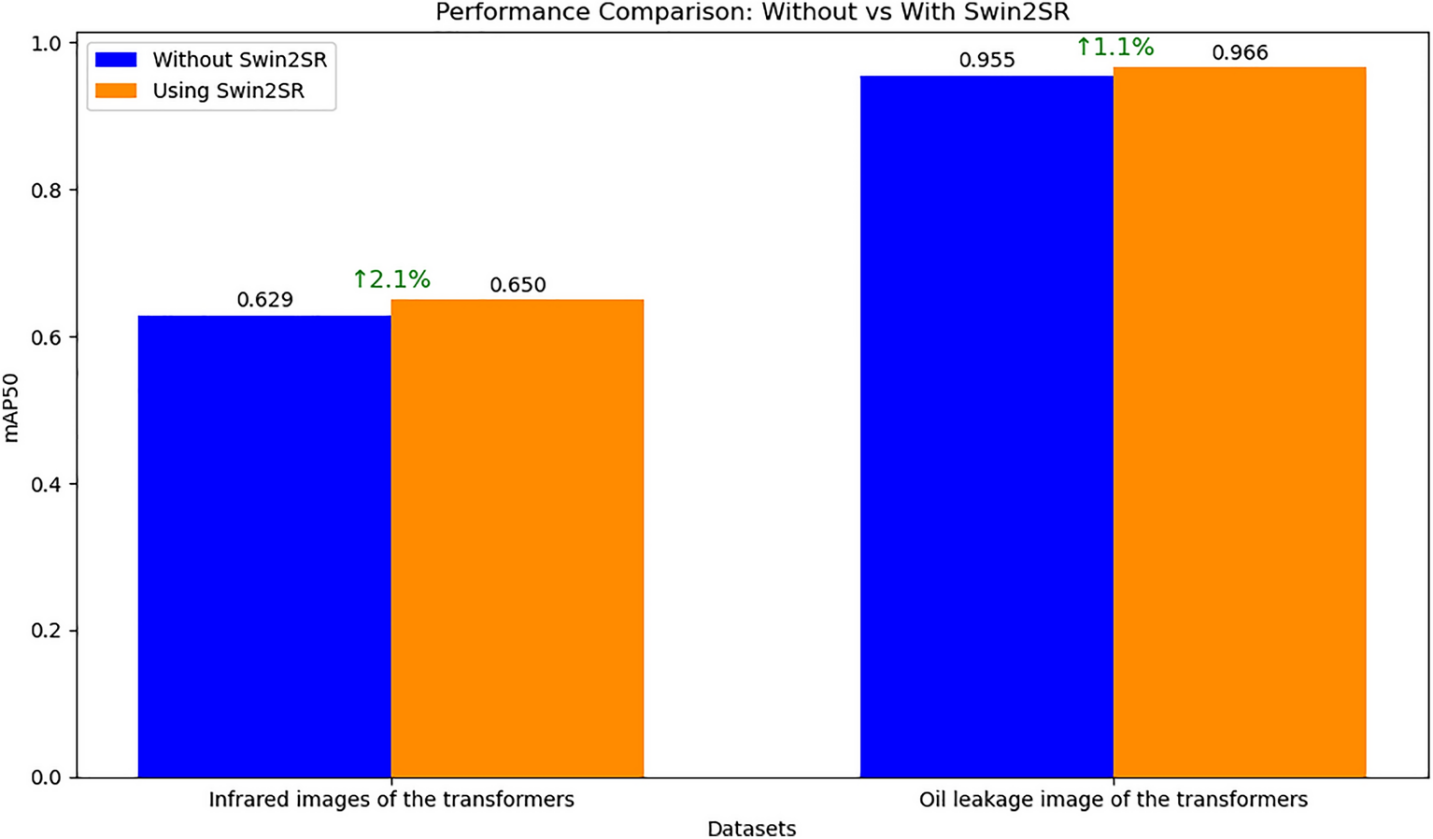
Comparison of Model Performance Before and After Swin2SR Data Augmentation.

The signal interference factors under industrial environments and remote network transmission conditions are complex and varied, which may lead to noise and blur in the acquired raw images to some extent. However, the quality of the images has a significant impact on model training and inference. High-quality images can represent more detailed and accurate global and local features. Therefore, preprocessing and augmentation of the raw data are essential in transformer monitoring tasks. In this study, the Swin2SR algorithm is applied to perform super-resolution reconstruction and detail feature reconstruction on the raw data, significantly enhancing the low-quality images from the industrial environment and providing a solid data foundation for precise model inference. To compare the positive impact of this module on the proposed DETR + X, inference models with and without Swin2SR data augmentation were separately trained and tested (Epoch = 50). The results, shown in Fig. 8, indicate that after applying Swin2SR for data augmentation, the model’s mAP50 score increased by 2.1% on the Infrared Images of the Transformers dataset, and by 1.1% on the Oil Leakage Image of the Transformers dataset. This demonstrates that the use of Swin2SR for enhancing low-quality data is effective.

##### Multi-scale attention features based on pyramid vision transformer (PVT)

**Fig. 9:**
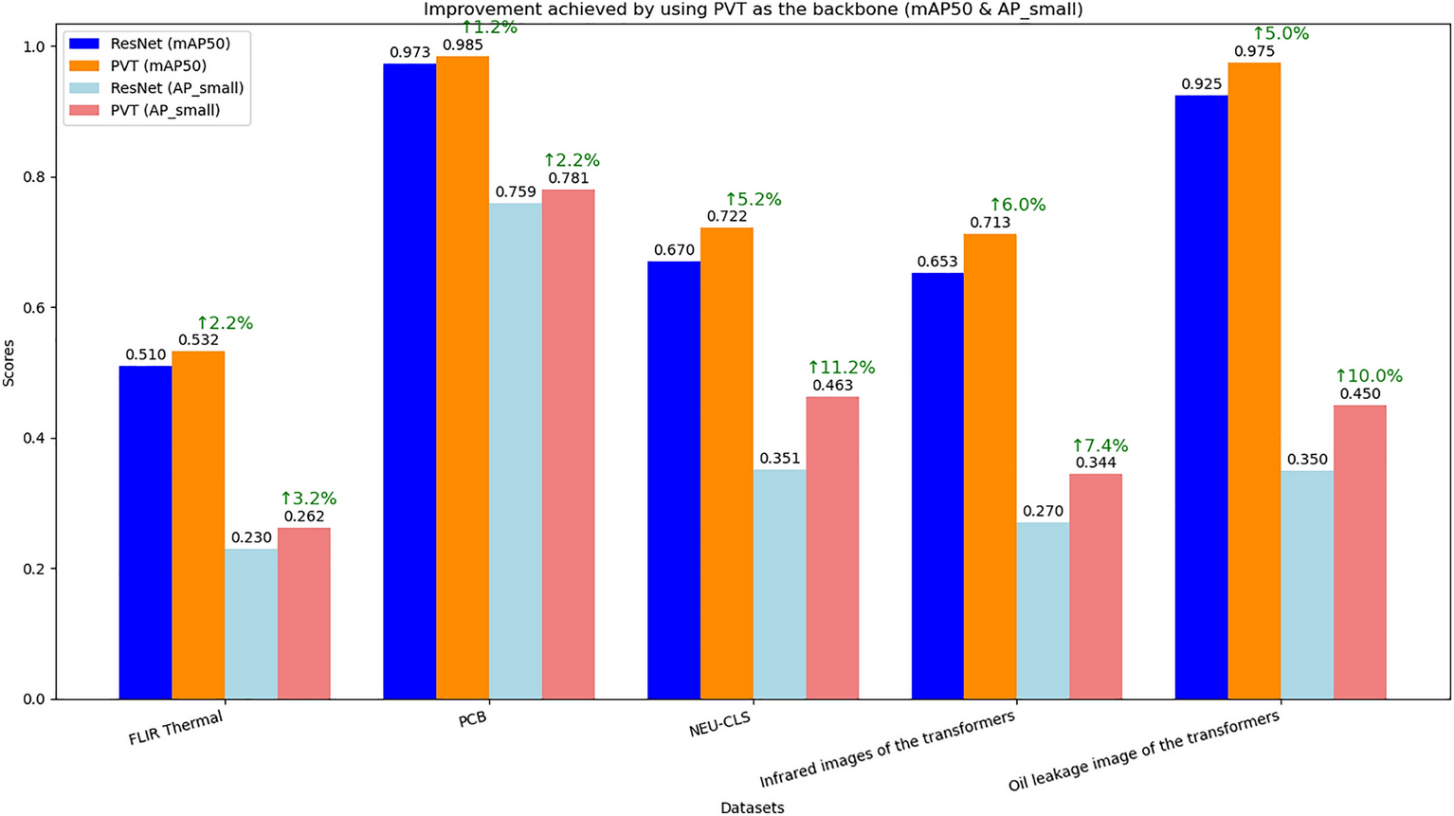
Comparison of Model Performance Before and After Adopting Pyramid Vision Transformer as the Feature Extraction Backbone.

The feature extraction backbone network of a detection model is crucial for its learning and inference performance. To validate the significant role of the Pyramid Vision Transformer (PVT) used in this study during feature extraction, the performance of the proposed DETR + X model with two different backbone networks— the advanced ResNet and the PVT used in this study— was compared. After multiple rounds of experimental analysis, the performance of the model with ResNet as the backbone network was used as the baseline. The model performance improvements before and after replacing the original feature extraction backbone network with Pyramid Vision Transformer (PVT) were evaluated. As shown in Fig. 9, after adopting Pyramid Vision Transformer (PVT), the model’s detection accuracy improved across all datasets, with a notable enhancement in small object detection performance. The main reason is that the structure of the ResNet backbone network is relatively fixed. As the network depth increases, the limitations of convolutional operations and local receptive fields reduce the efficiency of capturing global information, thus restricting the overall feature extraction capability. In contrast, the PVT network introduces a pyramid structure, consisting of multiple layers of transformer blocks. During image processing, the input image is divided into many small patches, which are processed through multiple transformer blocks. Different blocks extract features at different scales, providing flexibility to adapt to multi-scale tasks. This structure is better suited for recognition tasks involving deformations and scale inconsistencies, as it can capture long-range dependencies within the image, addressing the limitations of convolutional neural networks in capturing global information.

##### Deformable attention mechanism

**Fig. 10:**
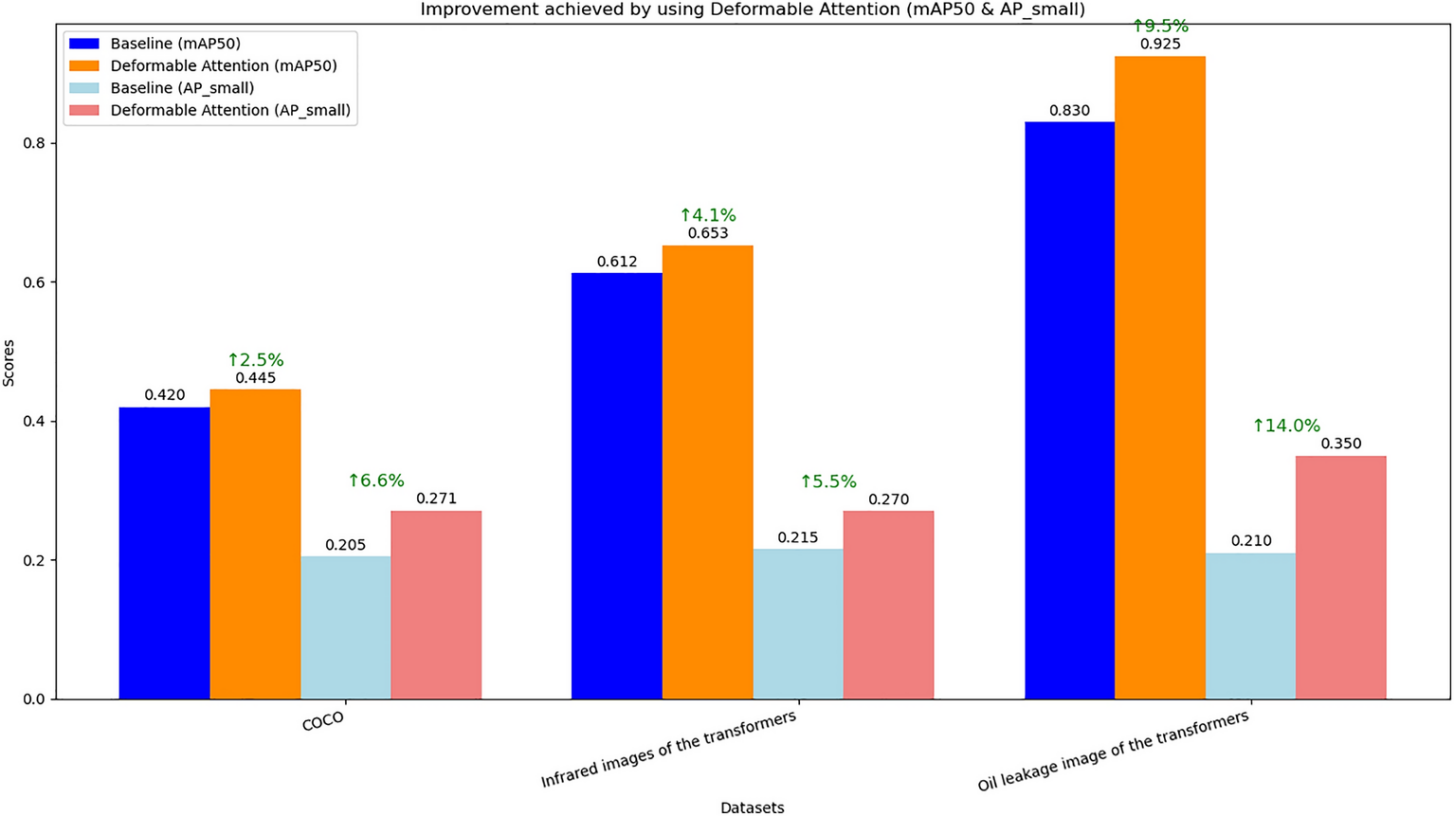
Comparison of Model Performance with Deformable Attention and Standard Attention.

To evaluate the contribution of the deformable attention mechanism to model performance, the model’s performance with deformable attention and the traditional standard attention mechanism was compared. The results are shown in Fig. 10. After adopting the deformable attention mechanism, the model’s detection accuracy showed a considerable improvement. The reason lies in the fact that the traditional self-attention mechanism is to establish global dependencies by calculating the similarity between each element in the input sequence and all other elements, and then updating the element representations through a weighted sum based on these similarities. However, compared to the traditional standard attention mechanism, the deformable attention mechanism can more selectively focus on key feature regions and perform feature fusion at different scales. This allows the model to better capture features at various scales, thereby enhancing its ability to represent complex patterns. At the same time, since it focuses only on relevant regions rather than computing attention for all positions, it consumes significantly less memory compared to the traditional standard attention mechanism.

##### DGA data modality transformation

By analyzing the experimental results (as shown in Table 5), it can be observed that when traditional computational methods are used as the classification backbone, the accuracy of transformer fault classification based on raw DGA data is limited. In traditional methods, the feature vector composed of the dissolved gas content in the oil is typically input into machine learning algorithms or convolutional networks for computation.Based on the DGA data modality transformation method proposed in this paper, the numerical data of multiple feature parameters are converted into RGB images. By utilizing state-of-the-art image classification backbone networks for transformer fault type classification, this approach not only integrates transformer infrared hotspot analysis, oil leakage analysis, and DGA gas data analysis into a unified computational framework, but also supports the input and inference of various heterogeneous modality information. After applying the DGA data modality transformation module based on the Gram angle field proposed in this paper, the accuracy of transformer fault classification using DGA data significantly improved, reaching nearly 100%. Compared to the model without the DGA data modality transformation module, there was a substantial increase in accuracy. These experimental results highlight the critical importance of the DGA data modality transformation module for transformer fault classification.

##### Intelligent decision-making module based on LLM (large language model)

**Fig. 11:**
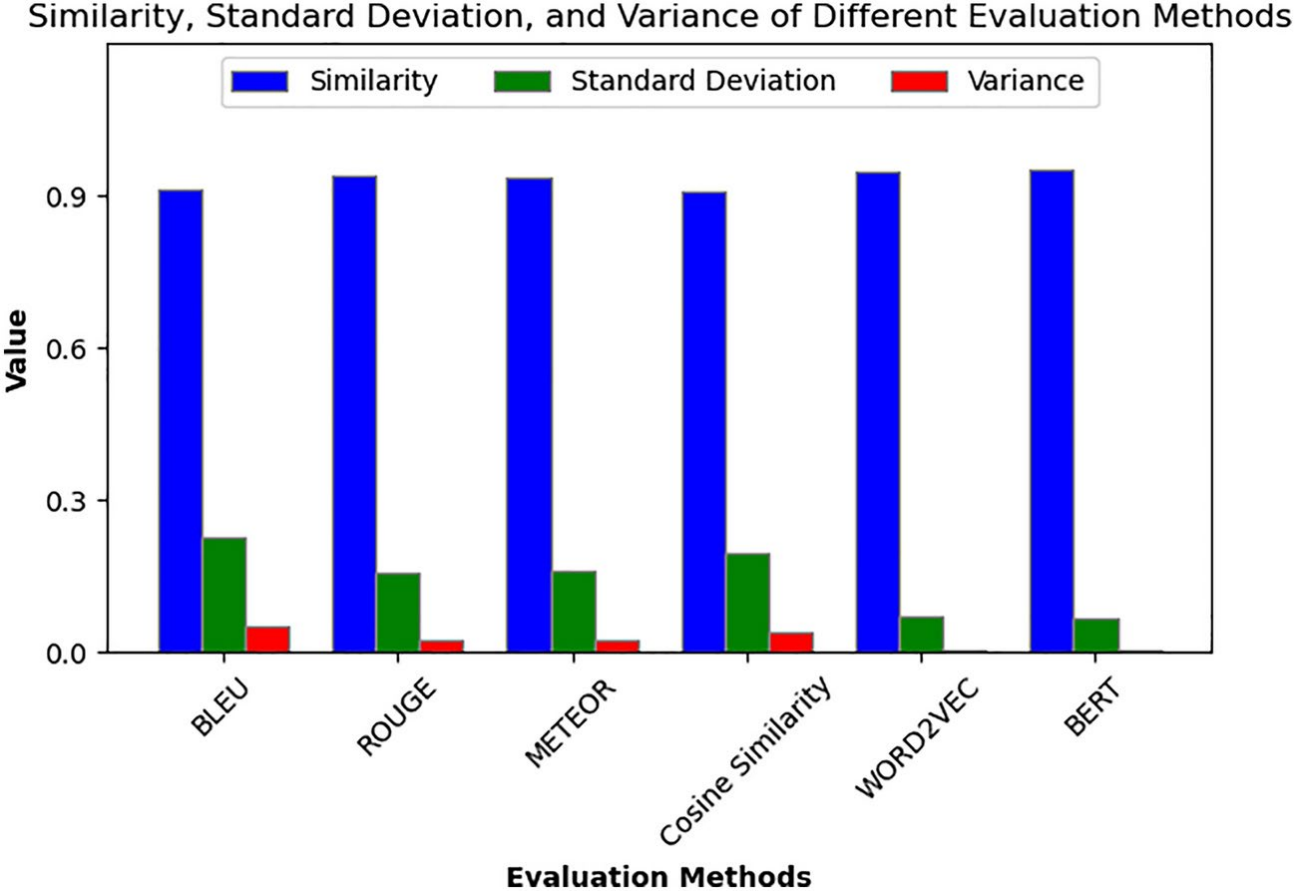
The performance of the model’s inference capability based on different evaluation metrics.

To evaluate the paradigm shift and significant contributions brought by this module to the intelligent operation and maintenance (O&M) of transformers, historical decision-making data, expert knowledge, and maintenance case studies related to transformer operation and maintenance were used as the corpus. Based on the LLAVA_7b model, the LLM model for transformer intelligent O&M decision-making was fine-tuned using the LoRA method. Subsequently, the analysis focuses primarily on two aspects: the accuracy of root cause analysis for abnormal phenomena and decision recommendation generation, as well as the response time from the occurrence of the anomaly to the completion of the decision. To evaluate the accuracy of decision text generation, various evaluation metrics including BLEU, ROUGE, METEOR, cosine similarity, WORD2VEC, and BERT were used to assess the similarity between the model’s inference results and the reference standards. As shown in Fig. 11, the similarity between the model-generated inference results and the standard reference texts exceeds 90% across all evaluation metrics. This indicates that the LLM-based intelligent decision-making module is capable of providing highly accurate root cause analysis for abnormal phenomena and decision recommendation text descriptions, which are of significant reference value for intelligent monitoring and maintenance activities of transformers.

In addition, to analyze the improvement in operational decision response efficiency after using the intelligent decision-making module, the decision response times under two scenarios—using the intelligent decision-making module and not using it—were statistically analyzed and systematically compared. As shown in Table 6, under the traditional model based on manual experience analysis, engineers need to review relevant reference materials and may also need to communicate and discuss with colleagues. They then rely on their personal expertise and experience to analyze and judge the causes of anomalies and the necessary actions to take. This process incurs a relatively long time cost. However, since the LLM-based expert system has learned all existing experiential knowledge and classic cases, and possesses intelligent reasoning and generalization capabilities, it can directly and in real-time generate high-quality analytical descriptions and decision recommendations. Additionally, it can engage in real-time human–machine dialogue with engineers, significantly improving the efficiency of operational decision-making response.

**Table 6:** Comparison of Analytical and Decision-Making Capabilities.

Method		Reviewing materials and communication	Decision generation	Human–machine dialogue
Based on manual experience	Junior engineer	30 min	20 min	/
	Senior engineer	15 min	10 min	/
LLM-based expert system	AI expert	No need	Second-level response	Instant Q&A

### Implementation of intelligent operation and maintenance for transformers

#### Experimental environment

**Fig. 12:**
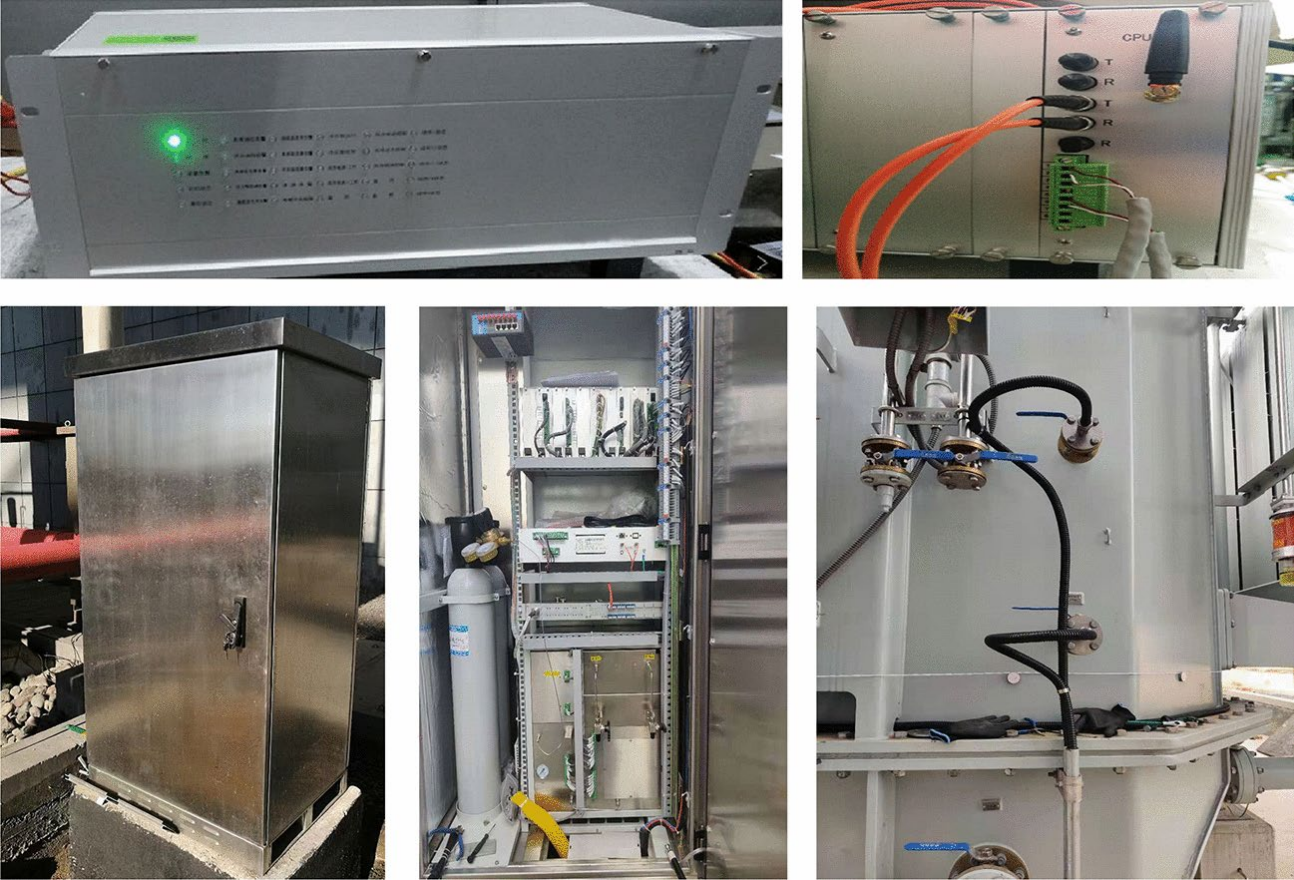
Oil Chromatography Data Collection Device and Installation Site.

**Fig. 13:**
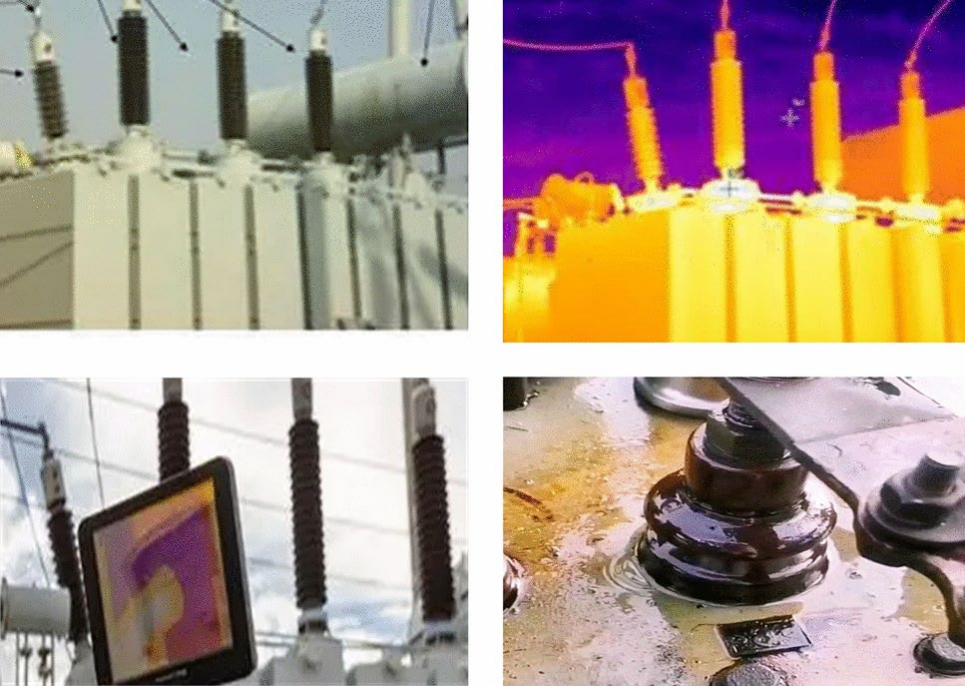
Acquisition of Infrared and Visible Light Images of Transformers.

To validate the method proposed in this paper, a digital twin platform for monitoring the operational status of transformers and facilitating intelligent operation and maintenance has been established. This platform is capable of collecting, transmitting, and transforming multimodal data related to transformer operations. As shown in Figs. 12 and 13, infrared thermal cameras, video surveillance cameras, and DGA data detection devices are deployed at the transformer operation site. Through a 5G network, the monitoring images and gas data are transmitted in real time to the back-end command center. The command center receives the remotely transmitted images and data, if the received data is DGA data, it is converted from one-dimensional DGA data to three-dimensional DGA feature maps using the Gram angle field transformation algorithm. Subsequently, these images are input into the previously trained DETR + X anomaly detection model for inference to analyze in real time whether the transformer exhibits issues such as abnormal hotspots, partial discharge, or oil leaks. Since the data collection devices are deployed at the equipment operation site, the collected images and DGA (Dissolved Gas Analysis) data need to be transmitted remotely over the network. When transmitting data through a 5G network, the transmission may be affected by factors such as high-frequency noise, multipath effects, and electromagnetic interference, which can introduce varying degrees of noise into the received images and data. This may result in issues such as signal distortion and image blur. To address these issues, this study employs a combination of denoising filters (such as Gaussian filters) and high-resolution reconstruction based on the Swin2SR model to preprocess the received data. This approach helps to remove noise from the original images and enhances their resolution, structural details, and textures, ultimately improving the quality and clarity of the images.

To evaluate the overall system response and the time requirements for monitoring and maintenance, extensive time cost testing was conducted. This includes measuring the time consumption from the initial data capture at the device collection end, through 5G network transmission, raw data preprocessing, image inference, and the entire decision-generation process. The key test parameters and results are shown in Table 7.

**Table 7:** System Response Time.

Parameter		Value
Network transmission time (denoted as *t*_1_)		≤ 200 ms
Data preprocessing time	Gaussian denoising (denoted as *t*_2_)	≤ 500 ms
	Swin2SR high-resolution reconstruction enhancement (denoted as *t*_3_)	≤ 1 s
DGA data conversion time (denoted as *t*_4_)		≤ 50 ms
Image inference time (denoted as *t*_5_)		≤ 3 s
Decision reasoning time (denoted as *t*_6_)		≤ 3 s

The temperature variation of a transformer is usually a gradual process. When the transformer experiences overload, aging, or other faults, the temperature increase typically does not change abruptly in a short period. Similarly, the concentration change of dissolved gases in transformer oil is also a progressive process. The accumulation of dissolved gases is often the result of a gradual evolution of abnormalities, such as under conditions of heated insulation oil or overcurrent, where gases gradually accumulate. These changes generally require a certain period of accumulation before reaching alarm thresholds or fault indicators. Under normal conditions, monitoring every 10 min is generally sufficient to meet the monitoring requirements. Once an anomaly is detected, the monitoring frequency can be increased, adjusting to every 3 min. Let *N* represent the monitoring frequency of the transformer’s status, *T*_*a*_ denote the unit response time for infrared image and oil leakage image monitoring tasks in the intelligent O&M system, and *T*_*b*_ represent the unit response time for dissolved gas monitoring in the oil. The system response time and the monitoring requirements for the transformer can be described as follows:

11 $$T_{a} = t_{{1}} + t_{{2}} + t_{{3}} + t_{{{5} + }} t_{{6}} \le {7}.{\text{7s}}$$

12 $$T_{b} = t_{{1}} + t_{{{5} + }} t_{{6}} \le {6}.{\text{2s}}$$

13 $$T_{a} < < N$$

14 $$T_{b} < < N$$

15 $$T_{a} + T_{b} < < N.$$

Based on the above experimental analysis, it can be concluded that the overall system response time of the proposed method fully meets the monitoring and operation-maintenance (O&M) requirements of transformers. By periodically monitoring infrared images, oil leakage images, and gas content, potential anomalies can be quickly detected, allowing for timely warnings. This provides relevant personnel with sufficient time and accurate solutions, effectively ensuring the safe operation and reliability of the transformer.

#### Application cases

**Fig. 14:**
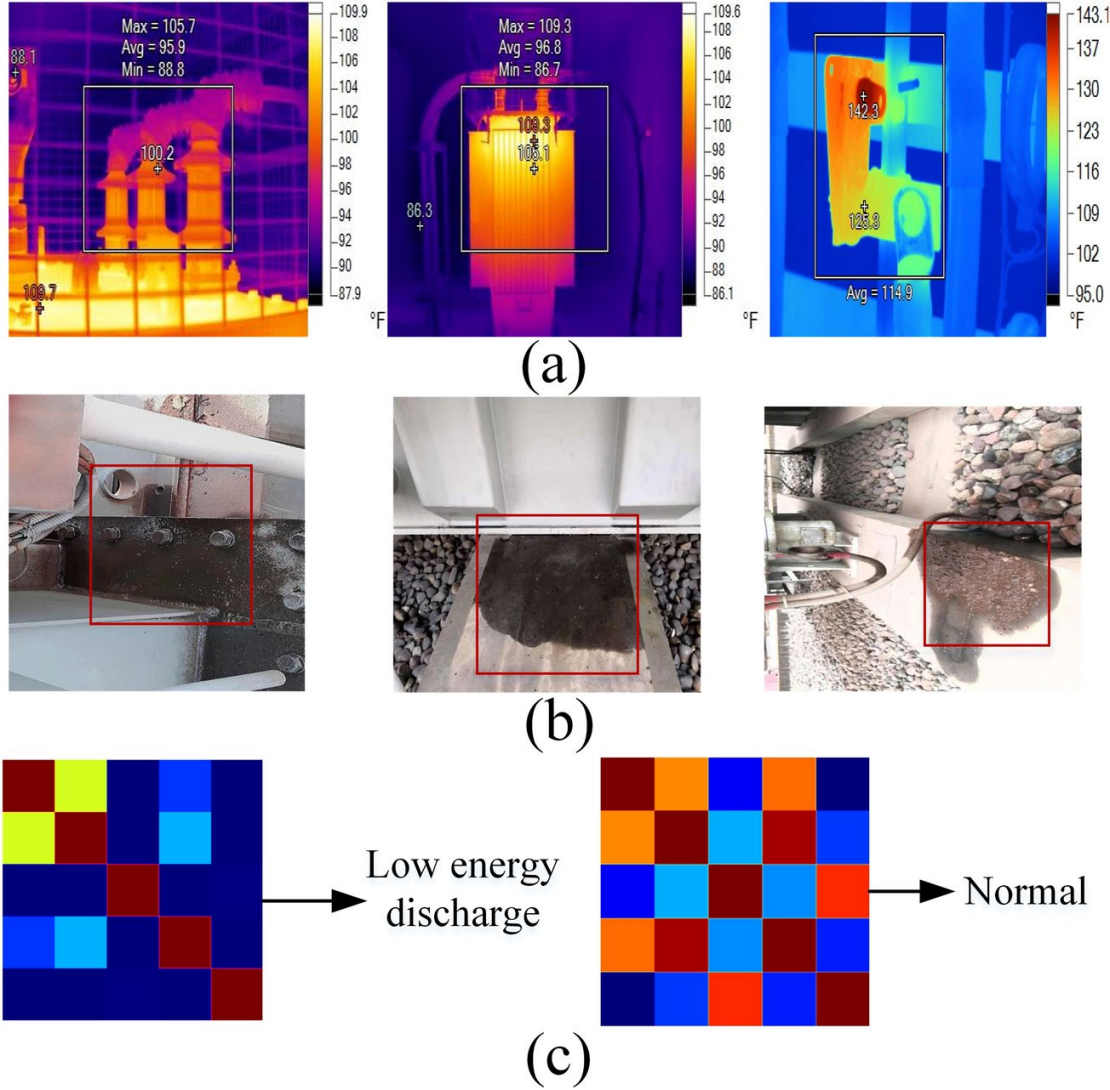
Identification of Transformer Operational Status Based on the DETR + X Visual Detection Large Model.

The digital twin intelligent operation and maintenance platform is deeply integrated with the DETR + X visual detection model proposed and trained in this study, employing the visual detection model as the brain for anomaly and fault identification in transformers. As illustrated in Fig. 14, this platform enables accurate and efficient recognition of the current operational status based on infrared images, visible light images, and DGA feature maps of the transformer. Utilizing the DGA feature maps, the platform can accurately identify conditions such as abnormal discharges and abnormal temperature rises in real time, while also coordinating and merging DGA analysis results with detection tasks from other modalities. For instance, upon detecting an abnormal temperature rise, the system immediately focuses on the detection results from infrared images, if abnormal hotspots are present, they are accurately annotated in the infrared images, displaying the hotspot temperature values in the corresponding image regions. Moreover, by analyzing and reasoning through leaked oil images, the system accurately identifies the existence of oil leakage faults, providing marked annotations and alerts indicating the location of leaks.

**Fig. 15:**
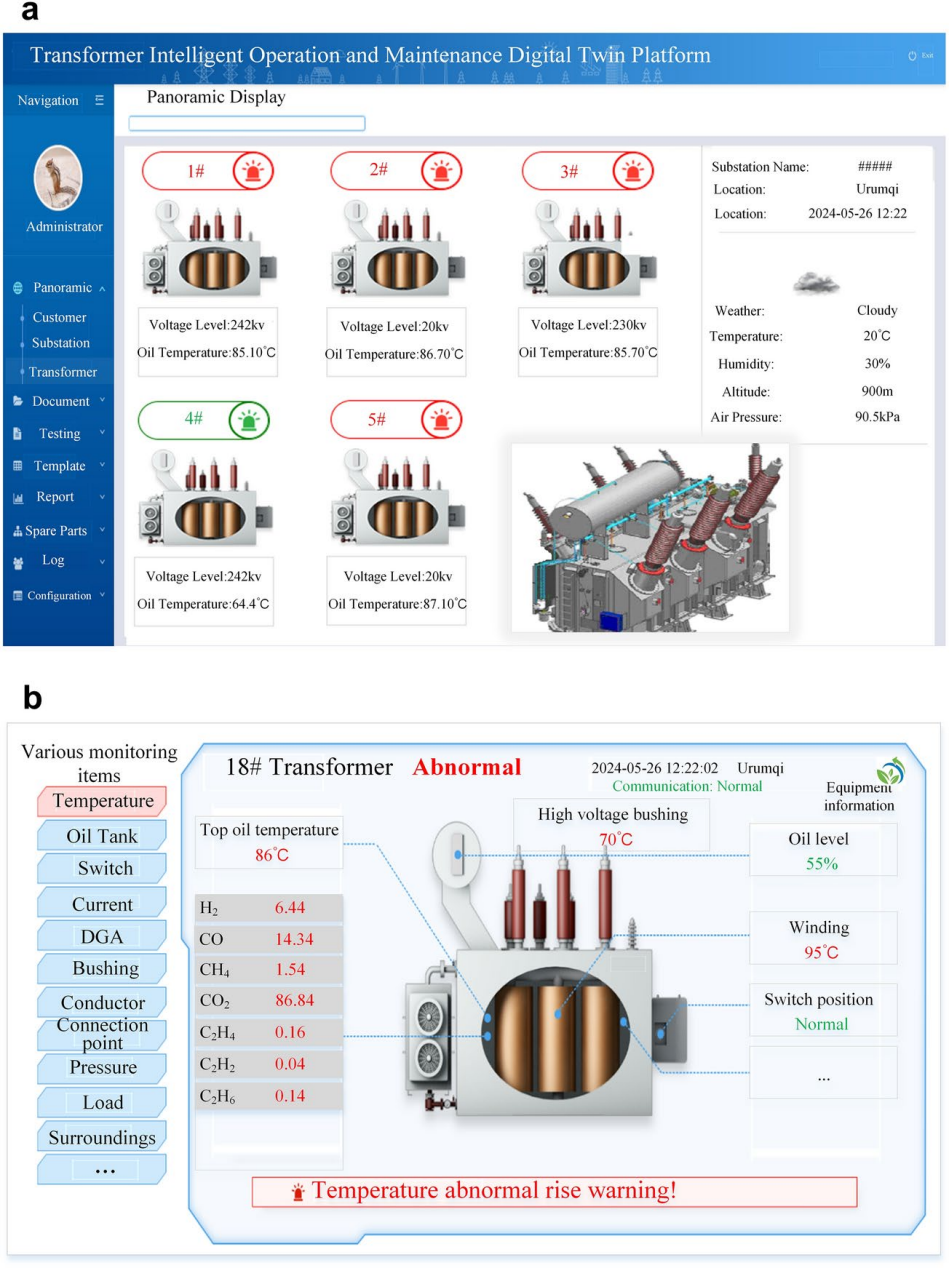
Real-Time Monitoring and Anomaly Warning of the Intelligent Operation and Maintenance Digital Twin Platform for Transformers.

Additionally, this digital twin intelligent operation and maintenance platform can perform real-time visual monitoring of the transformer’s operating environment and status, issuing timely alerts for any abnormal conditions. Figure 15 shows key parameters in real time, including the location of each transformer, weather conditions, rated voltage, oil temperature, component temperatures, switch positions, oil tank status, and gas data values. Based on the analysis results from the DETR + X visual detection model developed in this study, immediate fault alerts are issued if anomalies are detected, accompanied by detailed information about the abnormal equipment, with alert messages continuously displayed until the engineer has resolved the issue. Through the deep integration of digital twin technology and the DETR + X visual detection model, the workload of engineers is significantly reduced, enhancing the real-time, accuracy, and convenience of transformer operational status monitoring.

**Fig. 16:**
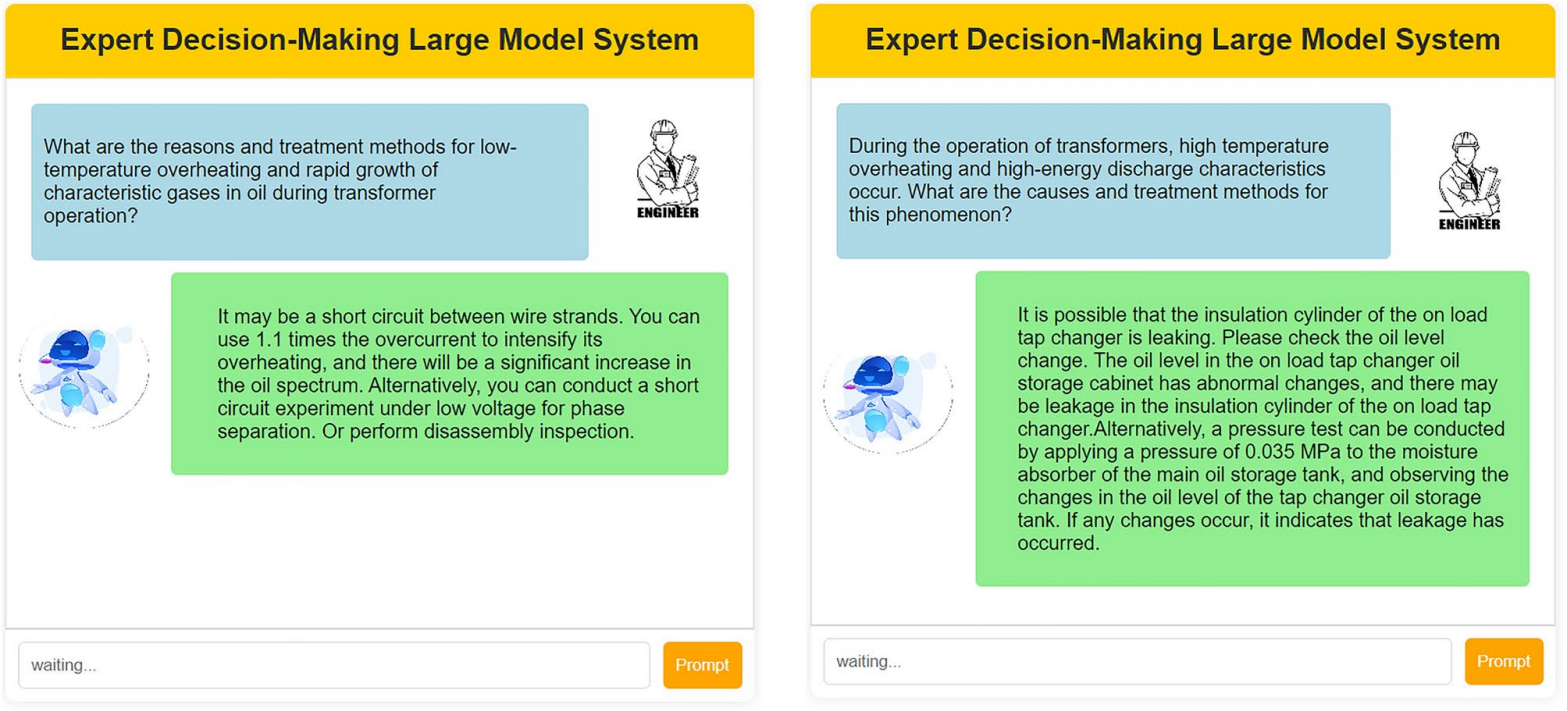
Intelligent Operation and Maintenance Decision Recommendations Enhanced by LLM.

Finally, based on historical decision data related to transformer operation and maintenance, expert knowledge, and repair cases, this study fine-tuned the LLAVA-7b model, which is utilized as an expert decision recommendation system for transformer operation and maintenance. Furthermore, an end-to-end integration of the previously proposed and trained DETR + X visual detection model with the LLAVA-7b model was achieved. When the DETR + X visual detection model identifies any anomalies or faults, the detection results are input into the LLAVA-7b model. Leveraging the language understanding and semantic reasoning capabilities of the LLAVA-7b model, it can swiftly and accurately analyze the root causes of the identified anomalies and provide guiding recommendations for maintenance based on various types of anomalies and faults. Maintenance personnel can then systematically investigate potential issues based on the recommended steps from the expert decision system, as illustrated in Fig. 16.

## Conclusion

The traditional monitoring and maintenance of transformer operations suffer from poor real-time responsiveness, limiting the ability to quickly address anomalies on-site and hindering the intelligent recognition of abnormal states through virtual-physical integration and visualization. To enhance the real-time efficient monitoring and intelligent operation and maintenance of transformer status, this study proposes a multi-modal and multi-scale intelligent operation and maintenance method based on the improved DETR + X deep visual large model and digital twin technology. By applying deep visual techniques to the detection and recognition of transformer operational status, we propose an enhanced DETR + X model, focusing on improvements in five areas: multi-dimensional data augmentation, multi-modal information fusion, multi-scale attention feature extraction, deformable attention mechanisms, and LLM-enhanced intelligent decision-making. Evaluations and tests were conducted on several publicly available datasets as well as the transformer dataset constructed in this study, with results indicating notable improvements in both object detection and image classification tasks. The model achieved the highest mAP50 scores and small object detection capabilities, with a classification accuracy of 100% for DGA feature maps. Compared to existing advanced methods, the performance reached an optimal level. Additionally, by deeply integrating the improved DETR + X deep visual model with digital twin, a real-time intelligent monitoring and maintenance system for transformer operation was developed, enabling multi-modal twin data collection, digital twin visualization, efficient status detection and recognition, and intelligent generation of operational decision recommendations.

Despite the feasible implementation methods and more accurate algorithm models provided by the aforementioned research for transformer operation monitoring and maintenance tasks, there remain areas that require further enhancement. On one hand, while the proposed model accommodates various modalities, it lacks effective alignment and collaborative learning between them. Future research could explore novel fusion frameworks to better integrate features from DGA feature maps, infrared images, oil leakage images, and other data types, enhancing multi-modal collaborative learning. On the other hand, although the model shows superior performance in detecting anomalies, it requires longer training and inference times, demanding higher GPU performance. Future efforts should focus on model compression and acceleration techniques, utilizing effective pruning and quantization methods to reduce the model’s size and inference time, thereby lowering training costs and improving inference immediacy.

## Data Availability

The datasets generated during and/or analysed during the current study are not publicly available due to some data involves related companies but are available from the corresponding author on reasonable request.
